# Comparative Assessment of Matrix Integration Dynamics Between Beetroot Powder and Paste in Wheat‐Based Cupcake Formulations

**DOI:** 10.1002/fsn3.70863

**Published:** 2025-09-07

**Authors:** Berlinda Agyei‐Poku, Sakyiwaa Afia Amponsah, Dedo Doreen Adi, Barikisu Mohammed

**Affiliations:** ^1^ Department of Hospitality and Tourism Sunyani Technical University Sunyani Ghana; ^2^ Department of Hospitality and Tourism Education Akenten Appiah‐Menka University of Skills Training and Entrepreneurial Development Kumasi Ghana

**Keywords:** composite flour, food fortification, functional food, nutritional enhancement, texture profile analysis

## Abstract

Beetroot ( *Beta vulgaris* L.) incorporation into cupcake formulations represents an innovative approach to developing functional bakery products that combine consumer appeal with enhanced nutritional value. This study investigated the incorporation of beetroot ( *Beta vulgaris* L.) as a functional ingredient in cupcakes. Beetroot was incorporated in two forms (powder and paste) at five concentration levels (10%–50% (w/w)) as partial substitutes for wheat flour. Physical properties and sensory attributes were evaluated. All physical properties showed strong linear relationships with beetroot concentration (*R* ^2^ > 0.95). Increasing beetroot concentration significantly increased hardness by 72.5% (powder) and 54.3% (paste) at maximum substitution level, while decreasing springiness by 19.6% (powder) and 14.4% (paste), cohesiveness by 29.5% (powder) and 23.4% (paste), and volume by 20.3% (powder) and 22.4% (paste). Redness (a) increased dramatically by 26.7‐fold (powder) and 29.0‐fold (paste), while lightness decreased by 42.6% (powder) and 45.8% (paste) at 50% substitution. Paste formulations consistently exhibited better textural properties, color development, and sensory acceptability compared to powder at equivalent concentrations. Sensory evaluation revealed that formulations containing 20% (w/w) beetroot powder and 30% beetroot paste received the highest acceptance scores (8.2 and 8.3 out of 9, respectively), slightly surpassing the control (8.0). Principal Component Analysis confirmed beetroot concentration as the primary factor influencing physical properties (82.4% variance), with form type as a significant secondary factor (12.7% variance). This study demonstrates that beetroot can be successfully incorporated into cupcakes at an acceptable level to create nutritionally enhanced bakery products with good physical and sensory characteristics.

## Graphical abstract

This study investigated the incorporation of beetroot in two forms (powder and paste) at five concentration levels (10%–50%) in cupcakes, finding strong linear relationships between beetroot concentration and physical properties. Paste formulations consistently outperformed powder at equivalent concentrations in textural properties, color development, and sensory acceptability, with optimal incorporation levels identified at 20% for powder and 30% for paste. Principal Component Analysis confirmed beetroot concentration as the primary factor influencing physical properties (82.4% variance), with form type as a significant secondary factor (12.7% variance), demonstrating that beetroot can be successfully incorporated into cupcakes at specific optimal levels to enhance nutritional value while maintaining acceptable quality attributes.

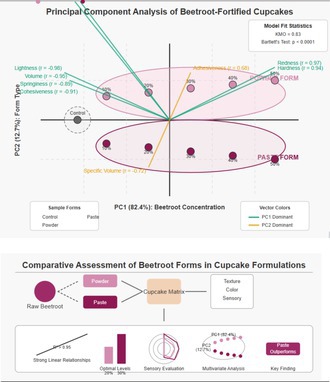

## 1 Introduction

The growing awareness of consumer demand for a health‐promoting diet has led to the development of functional foods in the food manufacturing industry. These foods not only satisfy hunger but also provide additional health benefits (Ergönül 2013). Bakery products, particularly cakes, are widely consumed across all age groups. However, they are typically high in carbohydrates and fats while being low in fiber and micronutrients (Gasparre et al. 2022). This nutritional profile makes traditional cakes less suitable for daily consumption from a health perspective (Gasparre et al. 2022).

*Beta vulgaris* L., commonly known as beetroot, offers significant potential as a functional ingredient due to its exceptional nutritional profile and unique bioactive compounds. Nutritionally, beetroot contains carbohydrates (8.8–10.2 g/100 g fresh weight), dietary fiber (2.0–3.2 g/100 g), folate (109 μg/100 g), potassium (325–400 mg/100 g), manganese (0.33 mg/100 g), and vitamin C (4.0–6.0 mg/100 g), while maintaining relatively low energy density (43–45 kcal/100 g) compared to other carbohydrate‐rich foods (Chhikara et al. 2019; Clifford et al. 2015). Beyond basic nutrients, beetroot is particularly rich in betalains (50–200 mg/100 g fresh weight), unique nitrogen‐containing pigments with demonstrated antioxidant, anti‐inflammatory, and hepatoprotective properties.

Recent advances in extraction and processing methodologies have demonstrated significant impacts on the functional properties of plant‐based ingredients. Wang et al. (2022) highlighted how different extraction approaches affect the physicochemical and antioxidant characteristics of fruit‐derived components, emphasizing the importance of processing method selection for effective bioactive retention. This principle directly applies to beetroot processing, where the choice between powder and paste preparation may significantly influence the final product's functional characteristics.

The growing awareness of consumer demand for health‐promoting diets has led to the development of functional foods in the food manufacturing industry. These foods not only satisfy hunger but also provide additional health benefits beyond basic nutrition (Ergönül 2013). Bakery products, particularly cakes, are widely consumed across all age groups due to their palatability, convenience, and cultural significance. However, traditional cakes are typically high in carbohydrates (45%–60% w/w) and fats (15%–25% w/w) while being low in dietary fiber (1%–3% w/w) and essential micronutrients, making them less suitable for daily consumption from a health perspective (Gasparre et al. 2022).

While beetroot has been incorporated into various food products including yogurt (Soutelino et al. 2023), probiotic drinks (Flores‐Mancha et al. 2021) ice cream, jelly, and cookies (Asadi and Khan 2021), limited research exists on its direct incorporation into cupcakes, particularly in Ghana. Furthermore, most studies have focused on beetroot powder, with minimal exploration of beetroot paste as an ingredient in baked goods.

The development of multifunctional ingredients capable of addressing multiple food quality parameters simultaneously represents a growing research focus. Khajeh et al. (2025) demonstrated how natural compounds can serve dual roles in food systems, providing both sensory enhancement and food safety benefits through reduced acrylamide formation. Similarly, beetroot incorporation may offer combined nutritional, sensory, and potentially protective effects in baked products.

This study aims to investigate the effect of incorporating beetroot in two forms (powder and paste) at varying concentrations (10%–50% (w/w)) on the physical properties and sensory attributes of cupcakes. This research addresses the gap in the literature regarding the use of beetroot in cupcakes, potentially offering a solution to the post‐harvest losses of beetroot in Ghana, which are estimated at approximately 17% (Mensah et al. 2021).

## 2 Materials and Methods

### 2.1 Materials

An all‐purpose wheat flour, margarine (Cook brand margarine), baking powder (sodium aluminum phosphate, CAS: 68476‐78‐8), granulated sugar (sucrose, CAS: 57‐50‐1), vanillin solution (food grade, 2% vanillin in ethanol, CAS: 121‐33‐5, Foster Clark's brand), were purchased from the Kumasi Central Market, Kumasi, Ghana. Fresh eggs and fresh beetroots ( *Beta vulgaris* L.) were acquired from the farmer at Agbogbloshie market in Accra. The beetroots were harvested during the dry season (December–February) at physiological maturity (8–10 weeks after planting) to ensure optimal betalain content and minimal moisture variability. Physical condition assessment revealed uniform shape (spherical to slightly elongated), absence of mechanical damage, and no signs of disease or pest infestation. All materials were securely transported in carrier bags to the sensory laboratory at the Food Research Institute of the Council for Scientific and Industrial Research (CSIR) in Accra.

#### 2.1.1 Equipment Specifications

Panasonic mixer grinder: Model MX‐AC300, 550W motor, speed range 10,000–18,000 rpm, stainless steel blades, Panasonic Corporation, Osaka, Japan.

Apex convection dryer: Model B35E, temperature range 40°C–80°C (±2°C accuracy), air circulation 2.5 m/s, capacity 10 kg, Apex Instruments Pvt. Ltd., Mumbai, India.

Conventional oven: Model KWS‐40A, temperature range 50°C–300°C (±5°C accuracy), Guangzhou Kaineng Electric Equipment Co., China.

Digital weighing balance: Model AS 220.R2, capacity 220 g, readability 0.1 mg, Radwag Balances and Scales, Poland.

Stable Micro Systems texture analyzer: TA‐XT plus Model 13051, 50 kg load cell, compression speed range 0.01–40 mm/s, Stable Micro Systems Ltd., Surrey, UK.

Konica Minolta colorimeter: CR‐400 series, Model NJ 07446, illuminant D65, 2° standard observer, measurement area 8 mm diameter, Konica Minolta Sensing Americas Inc., USA.

### 2.2 Experimental Design

The study employed a factorial design approach, consisting of two distinct forms of beetroot, specifically, powder and paste, tested across five varying concentration formulations: 10%, 20%, 30%, 40%, and 50% for each form. This resulted in a comprehensive 2 × 5 factorial design, culminating in a total sample size of ten. Additionally, a control sample composed entirely of 100% wheat flour was prepared for comparative analysis.

### 2.3 Processing of Beetroot

#### 2.3.1 Beetroot Paste Preparation

Fresh beetroots (2.5 ± 0.2 kg per batch) were thoroughly washed with potable water, peeled using stainless steel peelers, and diced into uniform pieces (8–10 mm^3^). The diced pieces were blended using a Panasonic mixer grinder (Model MX‐AC300, 550W motor, speed range 10,000–18,000 rpm, stainless steel blades, Panasonic Corporation, Osaka, Japan) without water addition under carefully controlled conditions to ensure consistency and quality. The grinding process was conducted at a speed of 15,000 ± 500 rpm for a processing time of 10.0 ± 0.5 min of continuous operation. Temperature monitoring was maintained at 25°C ± 3°C throughout the process, achieved by implementing 30‐s intervals every 2 min to prevent overheating. Each grinding cycle processed a batch size of 500 ± 25 g to maintain uniformity, and the final particle size was confirmed to be less than 0.5 mm through sieve analysis.

The resulting paste exhibited a smooth, homogeneous consistency with a moisture content of 87.2% ± 1.5%. Following preparation, the paste was immediately transferred to sterile containers and refrigerated at 4°C ± 1°C for a maximum of 24 h before use to maintain freshness and prevent microbial growth.

#### 2.3.2 Preparation of Beetroot Powder

Fresh beetroots were processed following standardized dehydration protocols to ensure reproducible quality characteristics. The pre‐treatment phase involved washing and peeling procedures identical to those used for paste preparation, followed by slicing to a thickness of 2.5 ± 0.2 mm using a commercial mandoline slicer. Slice uniformity was maintained at greater than 95% within ±0.3 mm tolerance to ensure consistent drying behavior.

The drying process was conducted under controlled parameters with a drying temperature of 65.0°C ± 2.0°C, monitored using calibrated thermocouples every 2 h to ensure temperature stability. The total drying time was 26.0 ± 1.0 h with an air velocity of 2.5 ± 0.2 m/s maintained throughout the process. The loading density was carefully controlled at 3.5 ± 0.3 kg/m^2^ to ensure uniform air circulation and heat transfer. The final moisture content achieved was 8.2% ± 0.8%, as determined by the standard oven method at 105°C.

Following dehydration, the dried beetroot slices underwent grinding and sieving processes to achieve the desired particle size. The grinding was performed at 18,000 ± 500 rpm for 5.0 ± 0.3 min, with 30‐s cooling intervals implemented to prevent heat buildup and potential degradation of bioactive compounds. The ground material was then passed through a 200 mesh sieve (75 μm aperture size, stainless steel, Laboratory Test Sieve, Endecotts Ltd., London, UK) to achieve a final particle size distribution with *D*₅₀ = 45 ± 8 μm. The final moisture content of beetroot powder was 4.2% ± 0.6% w/w (dry basis), equivalent to 4.0% ± 0.5% w/w (wet basis), as determined by the standard oven method at 105°C for 4 h (Guerrero et al. 2025) with triplicate measurements. The powder was stored in sealed aluminum pouches with desiccant packets at room temperature (25°C ± 2°C) and relative humidity less than 40% rather than refrigerated conditions because: (1) the low moisture content (4.2% w/w DB) eliminates microbial growth risks, (2) room temperature storage prevents condensation formation that could occur during temperature fluctuations in refrigerated storage, (3) desiccant packets maintain optimal water activity (aw < 0.3) for maximum shelf stability, (4) aluminum packaging provides excellent barrier properties against light and oxygen, and (5) controlled room temperature storage is more practical for industrial applications and maintains powder flowability characteristics.

The preservation of bioactive compounds during processing represents a critical consideration in functional food development. Following the principles outlined by Selahvarzi et al. (2021) who demonstrated the importance of gentle processing for maintaining antimicrobial and antioxidant properties in plant‐based ingredients, our paste preparation protocol minimized thermal exposure and mechanical stress to preserve beetroot's natural bioactive profile.

#### 2.3.3 Process Flow Documentation

**FIGURE 1:**
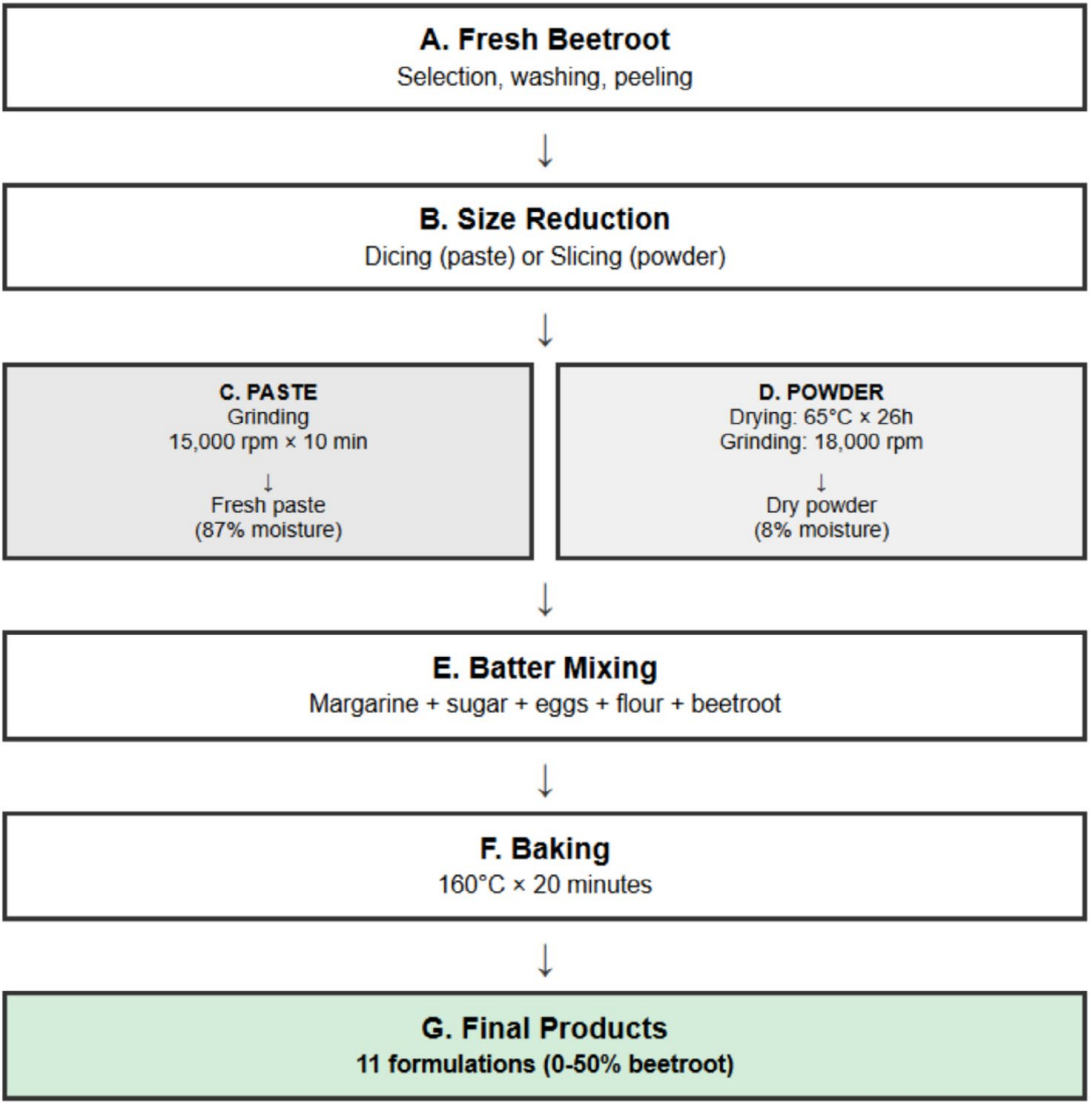
Sequential processing steps for beetroot‐fortified cupcake production.

Figure 1 provides a comprehensive visual documentation of the sequential processing steps employed in this study. The systematic approach ensures reproducibility and quality control throughout the production process.

### 2.4 Cupcake Preparation

The cupcake preparation followed a standardized protocol with all baking parameters representing mean values ± standard deviations from three independent batches (*n* = 3). The mixing process began with a creaming phase where margarine and sugar were combined for 5.0 ± 0.5 min at medium speed to achieve proper aeration and texture. Egg incorporation followed for 2.0 ± 0.3 min to ensure complete integration without overmixing. The dry ingredient folding process, including wheat flour, beetroot powder or paste, and baking powder, was conducted for 1.5 ± 0.2 min until just combined to avoid gluten overdevelopment. The final batter temperature was maintained at 22°C ± 2°C to ensure optimal consistency and handling properties.

Baking conditions were carefully controlled with an oven preheating time of 15.0 ± 2.0 min to ensure temperature stability before baking commenced. The baking temperature was maintained at 160.0°C ± 5.0°C, verified using an independent thermometer for accuracy, and the baking time was standardized at 20.0 ± 1.0 min. Each cupcake was prepared using a batter weight of 45 ± 2 g per mold, with standard cupcake liners having a 5.5 cm top diameter to ensure uniform size and baking characteristics.

**FIGURE 2:**
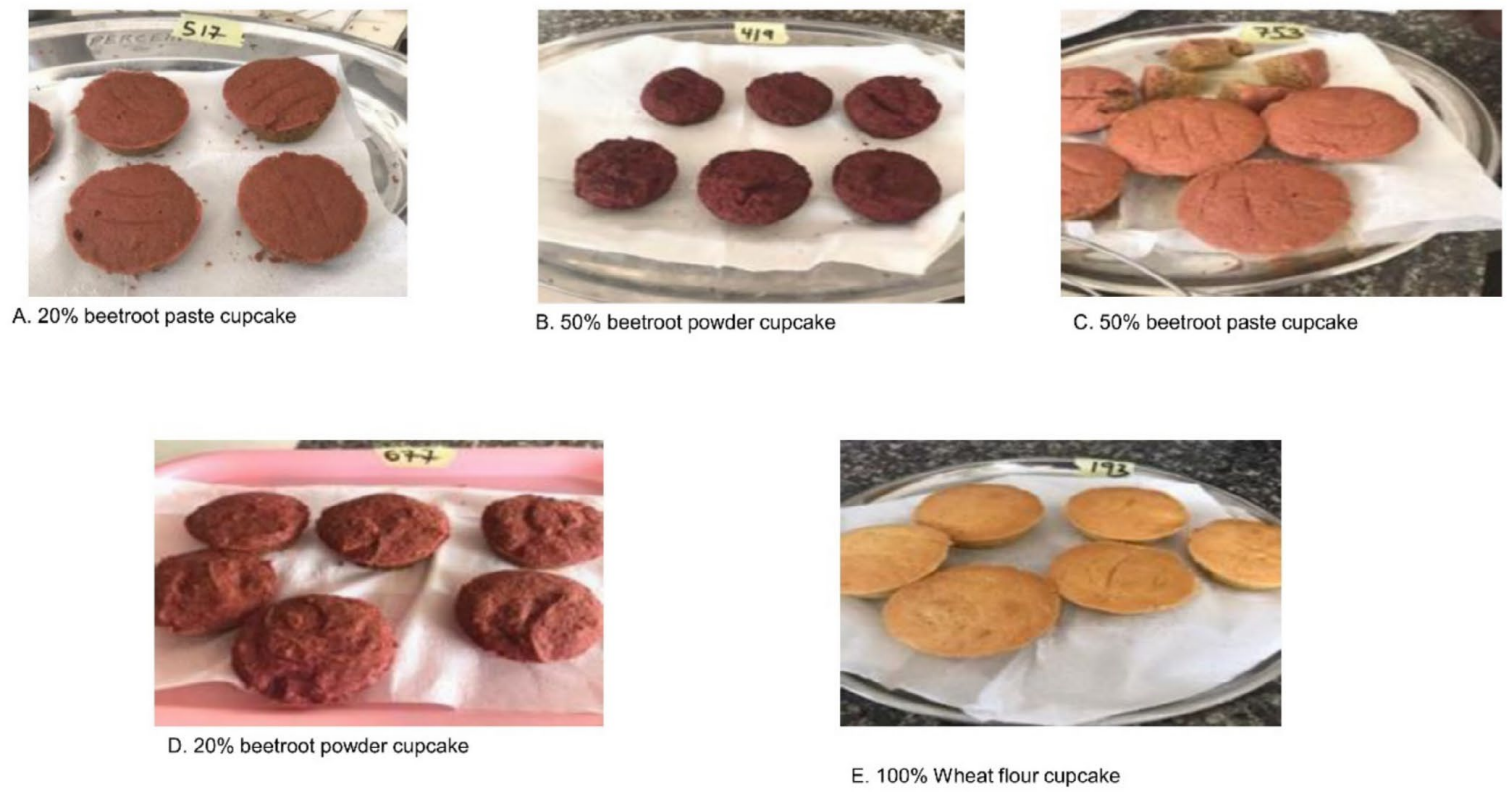
Visual appearance of beetroot‐fortified cupcakes demonstrating progressive color development with increasing beetroot concentration and form differences. (A) 20% w/w beetroot paste cupcakes displaying light reddish‐brown coloration with uniform distribution; (B) 50% w/w beetroot powder cupcakes exhibiting deep red‐brown color with more intense pigmentation; (C) 50% w/w beetroot paste cupcakes showing the most pronounced deep red colouration, demonstrating superior betalain retention; (D) 20% w/w beetroot powder cupcakes presenting moderate reddish‐brown color; and (E) 100% wheat flour control cupcakes maintaining traditional golden‐brown appearance.

The post‐baking protocol included a cooling time in molds of 10.0 ± 1.0 min to allow initial structure setting, followed by room temperature cooling for 45.0 ± 5.0 min at 25°C ± 2°C. The relative humidity during cooling was maintained at 65% ± 5% to prevent excessive moisture loss or absorption. Storage conditions until analysis were carefully controlled at 25°C ± 2°C and 60% ± 5% relative humidity for a maximum period of 6 h to ensure that analytical measurements reflected the fresh product characteristics without significant deterioration. The various cake formulations are presented in Table 1 and See Figure 2.

**TABLE 1:** (A) Beetroot powder (BRP) formulations. (B) Beetroot paste (BRT) formulations.

Ingredients	Control	BRP‐10%	BRP‐20%	BRP‐30%	BRP‐40%	BRP‐50%
(A)						
Wheat flour (g)	100	90	80	70	60	50
Sugar (g)	50	50	50	50	50	50
Margarine (g)	100	100	100	100	100	100
Eggs (number)	2	2	2	2	2	2
Vanilla essence (tsp)	1.25	1.25	1.25	1.25	1.25	1.25
Baking powder (tsp)	1.25	1.25	1.25	1.25	1.25	1.25
Beetroot powder (g)	0	10	20	30	40	50

Ingredients	Control	BRT‐10%	BRT‐20%	BRT‐30%	BRT‐40%	BRT‐50%
(B)						
Wheat flour (g)	100	90	80	70	60	50
Sugar (g)	50	50	50	50	50	50
Margarine (g)	100	100	100	100	100	100
Eggs (number)	2	2	2	2	2	2
Vanilla essence (tsp)	1.25	1.25	1.25	1.25	1.25	1.25
Baking powder (tsp)	1.25	1.25	1.25	1.25	1.25	1.25
Beetroot paste (g)	0	10	20	30	40	50

*Note:* All beetroot substitutions are expressed as percentage weight‐for‐weight (% w/w) replacement of wheat flour. The percentages indicate the proportion of wheat flour replaced by beetroot in each formulation.

Abbreviations: BRP = Beetroot Powder; BRT = Beetroot Paste.

### 2.5 Physical Properties Determination

#### 2.5.1 Analysis of Texture Profile

The texture profile analysis (TPA) of the cupcakes was conducted using a Stable Micro Systems texture analyzer (TA‐XT plus Model 13051, 50 kg load cell, compression speed range 0.01–40 mm/s, Stable Micro Systems Ltd., Surrey, UK) following the methodology outlined by Agrahar‐Murugkar et al. (2016) Cylindrical‐shaped samples with dimensions of 22.5 mm in diameter and 30 mm in height were extracted using a size 15 cork borer. A two‐bit compression test was done at a testing speed of 5 mm/s, a pre‐test speed of 1 mm/s, a post‐test speed of 5 mm/s, and a deformation rate of 75% using an aluminum cylindrical probe with a diameter of 75 mm. The data acquisition rate was set at 200 pulses per second (pps). The adhesiveness, hardness, cohesiveness, and springiness were evaluated using the Exponent software.

#### 2.5.2 Volume Measurement

The cupcake volume (V, mL) was determined through the rapeseed displacement method as outlined by Rosell et al. (2001). Other volume‐related parameters, such as the weight (W, g), length, width, and height (L, Wi, H: m) of the cupcakes, were measured.

#### 2.5.3 Color Measurement

The color of the cupcakes was measured using a Konica Minolta CR series colourimeter (CR‐400 series, Model NJ 07446, illuminant D65, 2° standard observer, measurement area 8 mm diameter, Konica Minolta Sensing Americas Inc., USA). The CIE Lab color system was used to measure L* (lightness), a* (redness/greenness), and b* (yellowness/blueness) values as described by Macdougall (2010). The colorimeter was calibrated using standard white and black tiles provided by the manufacturer before each measurement session.

### 2.6 Sensory Evaluation

#### 2.6.1 Ethical Considerations and Sensory Evaluation Protocol

##### 2.6.1.1 Ethics Approval

The sensory evaluation protocol was reviewed and approved by the Institutional Ethics Committee of the Council for Scientific and Industrial Research—Food Research Institute.

#### 2.6.2 Participant Consent and Safety

All panelists (*n* = 15) provided written informed consent after being comprehensively briefed about the complete scope of the research study. The briefing session covered detailed explanations of the study objectives and all procedures they would be required to perform during the sensory evaluation. Participants were thoroughly informed about potential risks and benefits associated with their participation, including any possible adverse reactions to the test products and the anticipated benefits to food science research. Confidentiality measures were explained in detail, ensuring participants understood how their personal information and responses would be protected and used only for research purposes. Each participant was clearly informed of their fundamental right to withdraw participation at any time during the study without consequence or need for justification, and they were provided with complete contact information for study coordinators to address any questions or concerns that might arise during or after their participation.

#### 2.6.3 Selection Criteria

The selection of panelists followed strict criteria to ensure the validity and reliability of the sensory evaluation results. All participants were required to be between 25 and 45 years of age to ensure adequate sensory acuity while maintaining consistency in physiological responses. A critical requirement was that participants had no known food allergies or sensitivities to any of the study ingredients, including wheat, eggs, margarine, sugar, vanilla, baking powder, or beetroot, to prevent adverse reactions and ensure safe participation. Additionally, participants could not have any medical conditions that might affect their taste or smell capabilities, such as respiratory infections, medication use that alters taste perception, or chronic conditions affecting chemosensory function. All selected panelists possessed previous experience with sensory evaluation procedures, with a minimum of 6 months of prior participation in similar studies to ensure familiarity with hedonic scaling and evaluation protocols. Finally, participants were required to demonstrate availability for the complete evaluation session, including pre‐evaluation training and the full sensory assessment, to maintain consistency in the evaluation process.

#### 2.6.4 Safety Protocols

Comprehensive safety protocols were implemented throughout the study to ensure participant wellbeing and data integrity. All samples were prepared strictly under Hazard Analysis and Critical Control Points (HACCP) guidelines (Fraqueza and Barreto 2014), with careful attention to hygiene, temperature control, and contamination prevention at every stage of preparation. Fresh preparation of all cupcake samples occurred on the evaluation day to ensure optimal quality and safety, with no samples prepared in advance or stored overnight. Temperature control was meticulously maintained throughout the evaluation process, with all samples served at 22°C ± 2°C to ensure both safety and optimal sensory perception. Individual serving portions were prepared and served to each participant to prevent any possibility of cross‐contamination between panelists, with separate utensils and serving dishes used for each participant. Emergency protocols were established and clearly documented for the unlikely event of allergic reactions, including immediate access to emergency medical services, availability of antihistamines, and trained personnel capable of administering first aid, ensuring participant safety remained the highest priority throughout the evaluation process.

#### 2.6.5 Sensory Analysis

A sensory evaluation was conducted using a nine‐point hedonic scale (9 = like extremely, 5 = neither like nor dislike, 1 = dislike extremely) to ascertain the degree of overall preference for the beetroot cupcakes. Fifteen semi‐trained panelists from the Food Research Institute in Accra assessed the products based on color, sponginess, taste, aroma, mouthfeel, appearance, and overall acceptability. Prior to the evaluation, the panelists participated in a 30‐min orientation session focusing on the sensory attributes of cupcakes and the application of the hedonic scale. The samples were presented in a random order and coded with three‐digit numbers. The panelists were provided with water and cucumber slices to clean their palates (Lawless and Heymann 2010).

### 2.7 Statistical Analysis

Two‐way analysis of variance (ANOVA) was conducted using the general linear model procedure to evaluate the main effects (beetroot form and concentration) and their interaction on physicochemical and sensory attributes. Prior to analysis, data were tested for normality using the Shapiro–Wilk test and for homogeneity of variances using Levene's test. When significant differences (*p* < 0.05) were detected, multiple comparison tests were performed using Tukey's Honestly Significant Difference (HSD) test to control for family‐wise error rate.

For sensory evaluation data, non‐parametric Friedman's test was applied followed by Wilcoxon signed‐rank tests with Bonferroni correction to account for the ordinal nature of hedonic scales. Internal consistency of sensory panel responses was assessed using Cronbach's alpha coefficient.

Linear regression models were developed to quantify the relationships between beetroot concentration and physical properties, with the coefficient of determination (*R* ^2^) and root mean square error (RMSE) calculated to evaluate the model's fitness. Residual analysis was performed to verify regression assumptions.

Principal Component Analysis (PCA) was conducted on standardized (mean‐centred and variance‐scaled) data to identify latent patterns among variables. Kaiser‐Meyer‐Olkin measure and Bartlett's test of sphericity were used to confirm sampling adequacy for PCA. Component selection was based on eigenvalues > 1.0, and varimax rotation was applied to enhance interpretability.

Correlation analysis was conducted between instrumental measurements and sensory attributes using Pearson's correlation coefficient for parametric data and Spearman's rank correlation for non‐parametric data. Statistical power analysis was performed post hoc to confirm an adequate sample size for detecting meaningful differences (*β* = 0.8, *α* = 0.05).

All statistical analyses were performed using IBM SPSS Statistics (version 26, IBM Corp., Armonk, NY) and R statistical software (version 4.1.0, R Foundation for Statistical Computing, Vienna, Austria) with the ‘factoextra’ and ‘FactoMineR’ packages for multivariate analyses. Statistical significance was established at *p* < 0.05 for all tests.

All physical property measurements were conducted in triplicate (*n* = 3), while sensory evaluation involved 15 trained panelists (*n* = 15) with each sample evaluated once per panelist.

## 3 Results and Discussion

### 3.1 Physical Properties of Beetroot‐Fortified Cupcakes

#### 3.1.1 Textural Profile Analysis: Effect of Beetroot Concentration and Form

The textural properties of the beetroot‐fortified cupcakes are presented in Table 2. Hardness, which represents the force required to compress the food between the teeth, increased significantly (*p* < 0.05) with increasing beetroot concentration in both powder and paste forms. However, cupcakes fortified with beetroot paste exhibited lower hardness values compared to those containing beetroot powder at equivalent substitution levels. Generally, the texture of the paste formulations was softer, preserving springiness and cohesiveness better than powder at the same concentrations.

**TABLE 2:** Textural properties of beetroot‐fortified cupcakes.

Sample	Hardness (N)	Adhesiveness (N‐S)	Springiness	Cohesiveness
Control	18.23 ± 1.02ᵃ—	−0.08 ± 0.01ᵃ	0.92 ± 0.02ᵃ	0.78 ± 0.03ᵃ
BRP‐10%	19.45 ± 0.95ᵃᵇ	−0.10 ± 0.02ᵃᵇ	0.89 ± 0.03ᵃᵇ	0.76 ± 0.02ᵃᵇ
BRP‐20%	21.37 ± 1.10ᵇᶜ	−0.12 ± 0.01ᵇ	0.87 ± 0.02ᵇᶜ	0.73 ± 0.04ᵇ
BRP‐30%	24.56 ± 1.23ᶜᵈ	−0.15 ± 0.03ᶜ	0.84 ± 0.04ᶜᵈ	0.68 ± 0.03ᶜ
BRP‐40%	27.82 ± 1.32ᵈᵉ	−0.18 ± 0.02ᶜᵈ	0.79 ± 0.03ᵈᵉ	0.62 ± 0.05ᶜᵈ
BRP‐50%	31.45 ± 1.45ᵉ	−0.22 ± 0.04ᵈ	0.74 ± 0.05ᵉ	0.55 ± 0.04ᵈ
BRT‐10%	18.78 ± 0.88ᵃ	−0.09 ± 0.01^a^	0.90 ± 0.02ᵃᵇ	0.77 ± 0.03ᵃᵇ
BRT‐20%	20.14 ± 1.05ᵇ	−0.11 ± 0.02ᵃᵇ	0.88 ± 0.03ᵃᵇᶜ	0.74 ± 0.02^b^
BRT‐30%	22.67 ± 1.18ᵇᶜ	−0.13 ± 0.02ᵇᶜ	0.86 ± 0.02ᵇᶜ	0.71 ± 0.04ᵇᶜ
BRT40%	25.32 ± 1.27ᶜᵈ	−0.16 ± 0.03ᶜ	0.82 ± 0.04ᶜᵈ	0.65 ± 0.03ᶜ
BRT‐50%	28.97 ± 1.36ᵈᵉ	−0.19 ± 0.04ᶜᵈ	0.77 ± 0.05ᵈᵉ	0.59 ± 0.05ᶜᵈ

*Note:* The values shown are means ± standard deviation derived from three replicate measurements. Within each column, values marked with different superscript letters indicate statistically significant differences (*p* < 0.05).

Abbreviations: BRP = Beetroot Powder; BRT = Beetroot Paste.

**FIGURE 3:**
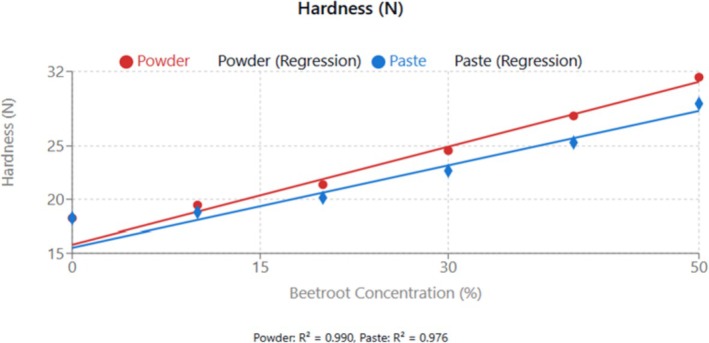
Effect of beetroot concentration (% w/w) on hardness (N) of cupcakes. Data points represent means ± standard deviations from three independent experiments (*n* = 3). Linear regression equations: Powder formulation: *y* = 0.304*x* + 15.795 (N), *R* ^2^ = 0.990; Paste formulation: *y* = 0.256*x* + 15.508 (N), *R* ^2^ = 0.976. Error bars indicate standard deviations.

The observed textural modifications align with findings by Wang et al. (2022) who reported that fiber‐rich plant extracts significantly influence the mechanical properties of food matrices through water‐binding interactions and protein network disruption. The optimization of processing parameters becomes crucial for maintaining acceptable textural attributes while maximizing functional benefits (see Figure 3).

##### 3.1.1.1 Hardness

**FIGURE 4:**
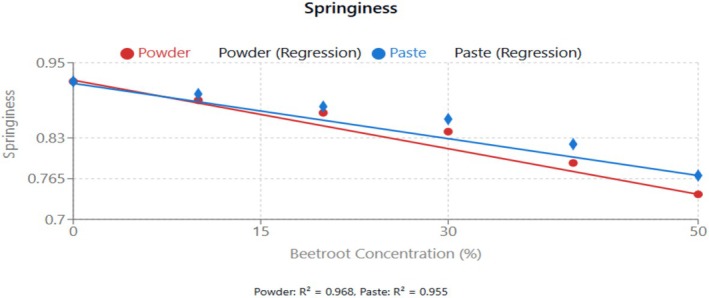
Effect of beetroot concentration (% w/w) on springiness (dimensionless) of cupcakes. Data points represent means ± standard deviations from three independent experiments (*n* = 3). Linear regression equations: powder formulation: *y* = −0.0036*x* + 0.922, *R* ^2^ = 0.968; paste formulation: *y* = −0.0029*x* + 0.917, *R* ^2^ = 0.955. Error bars indicate standard deviations.

From Figure 4, there was a linear increase in beetroot concentration from both forms (*R* ^2^ > 0.97). The rate of increase was higher for powder (0.304 N per 1% increase) compared to paste (0.256 N per 1% increase). As beetroot concentration increases in the formulation, the hardness of the cupcake also increases.

Beetroot contains significant amounts of insoluble dietary fiber, primarily composed of cellulose, hemicellulose, and lignin (Krapivnytska et al. 2022). These fibrous components create a more rigid structural network within the cupcake matrix, contributing to increased resistance to compression. The insoluble fiber particles act as reinforcing agents, similar to how aggregate materials strengthen concrete structures (Soleimani et al. 2018).

Beetroot is rich in pectin (0.5%–1.2% fresh weight), a structural polysaccharide that forms gel‐like networks in the presence of water and sugar (Krapivnytska et al. 2022). During baking, pectin undergoes thermal modification and cross‐linking, creating additional structural bonds that increase the firmness of the final product (Liu et al. 2020). The pectin's high water‐binding capacity also affects moisture distribution, leading to a denser, firmer texture.

Again, there are natural sugars in beetroot. These sugars participate in Maillard reactions during baking, forming protein‐sugar complexes that contribute to structural rigidity (Saeki et al. 2012). The additional sugar content from beetroot enhances caramelization reactions, producing firmer gel structures that increase overall hardness.

The interaction between wheat gluten and beetroot fiber components forms complex networks that alter the viscoelastic properties of the cupcake structure (Djordjević et al. 2021). These protein‐fiber interactions create additional cross‐links that resist deformation, manifesting as increased hardness.

Even in paste form, beetroot retains microscopic cellular fragments that act as discrete particles within the cupcake matrix (Mattar 2019). These particles create stress concentration points and disrupt the continuity of the gluten network, leading to a more heterogeneous structure with increased resistance to compression. These particles create stress concentration points and disrupt the continuity of the gluten network, leading to a more heterogeneous structure with increased resistance to compression (Mattar 2019). The incorporation of natural ingredients into pastry formulations has been shown to influence the hardness of the final product significantly. Various studies indicate that increasing the concentration of these ingredients often correlates with enhanced hardness, which can be attributed to their fiber content and structural properties. The addition of banana peels, for instance, increased the hardness of biscuits and muffins. While increased hardness may affect texture perception, formulations with moderate levels of natural ingredients often maintain improved sensory acceptability, balancing health benefits with consumer preferences (Aishah et al. 2020).

Springiness, which indicates how well a product physically bounces back after compression, went down significantly (*p* < 0.05) with increasing beetroot concentration. Generally, natural powders such as lotus leaf, lotus root, garlic, psyllium, broccoli, and cabbage have an impact on the quality characteristics of sponge cakes (Lu et al. 2010; Masoodi et al. 2002; Prokopov et al. 2015). These ingredients influence the cake's texture, volume, and sensory attributes, with varying effects on sponginess depending on the concentration and type of additive. This reduction in springiness could be attributed to the dilution of gluten in the wheat flour, which is responsible for the elastic properties of baked products. These ingredients tend to decrease the springiness of cakes. In most of these additives, the decrease was attributed to the powder absorption properties, which affect the cake's moisture and texture (Prokopov et al. 2015). However, psyllium seed mucilage at (0.25%) incorporation in cake increased springiness and overall texture as mucilages help maintain moisture and delay staling, thus contributing to springiness (Beikzadeh et al. 2020). As shown in Figure 4, there was a linear decrease in springiness with increasing beetroot concentration. The paste formulation maintained better springiness compared to the powdered one at equivalent concentrations.

**FIGURE 5:**
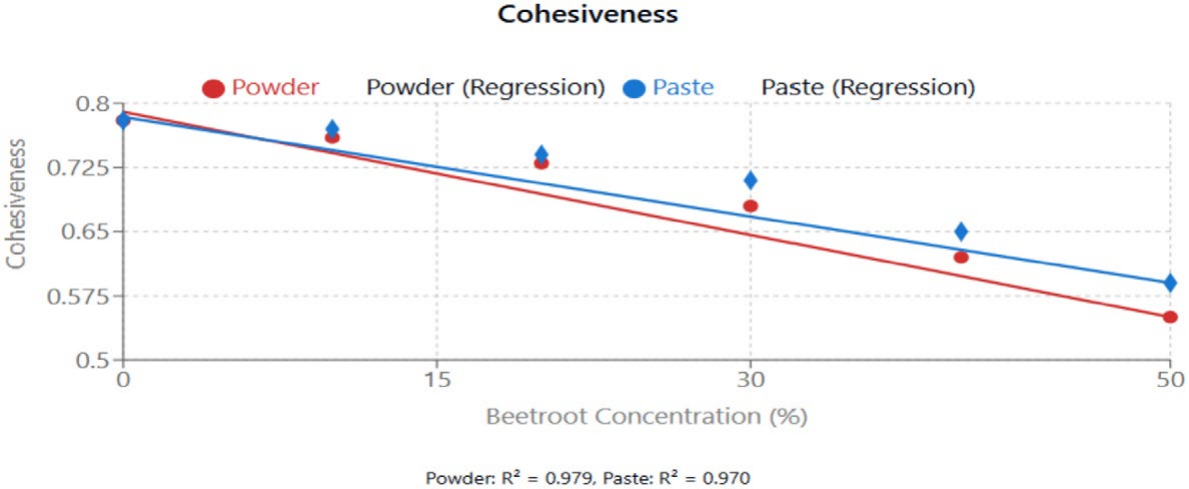
Effect of beetroot concentration (% w/w) on cohesiveness (dimensionless) of cupcakes. Data points represent means ± standard deviations from three independent experiments (*n* = 3). Linear regression equations: Powder formulation: *y* = −0.0048*x* + 0.790, *R* ^2^ = 0.979; Paste formulation: *y* = −0.0039*x* + 0.784, *R* ^2^ = 0.970. Error bars indicate standard deviations.

Cohesiveness, representing the strength of internal bonds making up the body of the product, also decreased significantly (*p* < 0.05) with increasing beetroot concentration. The reduction in cohesiveness in Figure 5 suggests that the incorporation of beetroot weakened the internal structure of the cupcakes, making them more susceptible to crumbling. The addition of a non‐gluten component in composite flours disrupts the viscoelastic properties of the gluten network (Herranz et al. 2016).

#### 3.1.2 Volumetric Characteristics and Mass Distribution

The volume and weight characteristics of the beetroot‐fortified cupcakes are presented in Table 3. A significant decrease (*p* < 0.05) in volume was observed with increasing beetroot concentration in both powder and paste forms. The control sample exhibited the highest volume (85.67 mL), while the 50% beetroot powder sample had the lowest volume (68.23 mL). Generally, paste formulations showed slightly lower volume and specific volume compared to powder at the same concentrations, potentially due to higher moisture content affecting the gluten network development.

**TABLE 3:** Volume and weight characteristics of beetroot‐fortified cupcakes.

Sample	Weight (g)	Volume (mL)	Specific volume (mL/g)
Control	52.34 ± 1.15ᵃ	85.67 ± 2.32ᵃ	1.64 ± 0.05ᵃ
BRP‐10%	53.12 ± 1.23ᵃᵇ	82.45 ± 2.18ᵃᵇ	1.55 ± 0.04ᵃᵇ
BRP‐20%	54.37 ± 1.32ᵃᵇ	79.32 ± 2.05ᵇᶜ	1.46 ± 0.06ᵇᶜ
BRP‐30%	55.89 ± 1.41ᵇᶜ	75.18 ± 1.98ᶜᵈ	1.34 ± 0.05ᶜᵈ
BRP‐40%	57.45 ± 1.53ᶜᵈ	71.56 ± 1.87ᵈᵉ	1.25 ± 0.04ᵈᵉ
BRP‐50%	59.23 ± 1.68ᵈ	68.23 ± 1.75ᵉ	1.15 ± 0.06ᵉ
BRT‐10%	70.89 ± 1.71ᵇ	81.10 ± 0.82ᵇ	1.51 ± 0.07ᵇᶜ
BRT‐20%	63.45 ± 1.57ᶜ	77.82 ± 1.25ᶜ	1.41 ± 0.06ᶜᵈ
BRT‐30%	55.78 ± 1.43ᵈᵉ	73.45 ± 1.53ᵈᵉ	1.29 ± 0.07ᵈᵉ
BRT‐40%	48.23 ± 1.32ᵉᶠ	69.87 ± 1.53ᵈᵉ	1.19 ± 0.05ᵈᵉ
BRT‐50%	42.56 ± 1.18ᶠᵍ	66.45 ± 2.05ᶠᵍ	1.09 ± 0.04ᶠᵍ

*Note:* The values shown are means ± standard deviation of three separate determinations. Statistical significance (*p* > 0.05) between values within the same column is indicated by different superscript notations.

Abbreviations: BRP = Beetroot Powder; BRT = Beetroot Paste.

The decrease in volume can be attributed to the dilution of gluten proteins, which are responsible for forming the viscoelastic network that entraps gas during fermentation and baking, resulting in the characteristic volume of baked products (Herranz et al. 2016). As the proportion of beetroot increased, the proportion of gluten‐forming proteins decreased, resulting in reduced gas retention and, consequently, a lower volume.

**FIGURE 6:**
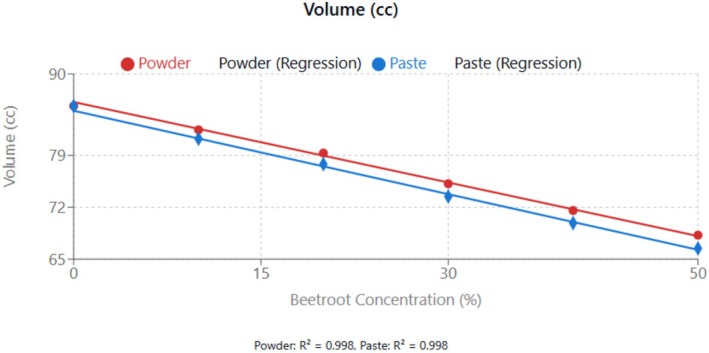
Effect of beetroot concentration (% w/w) on volume (mL) of cupcakes. Data points represent means ± standard deviations from three independent experiments (*n* = 3). Linear regression equations: powder formulation: *y* = −0.362*x* + 86.208 (mL), *R* ^2^ = 0.998; paste formulation: *y* = −0.375*x* + 85.033 (mL), *R* ^2^ = 0.998. Error bars indicate standard deviations.

From Figure 6, there was a linear decrease in cupcake volume with increasing beetroot concentration for both powder and paste. The rate of decrease was slightly greater for paste (−0.375 mL per 1% increase) compared to powder (−0.362 mL per 1% increase).

**FIGURE 7:**
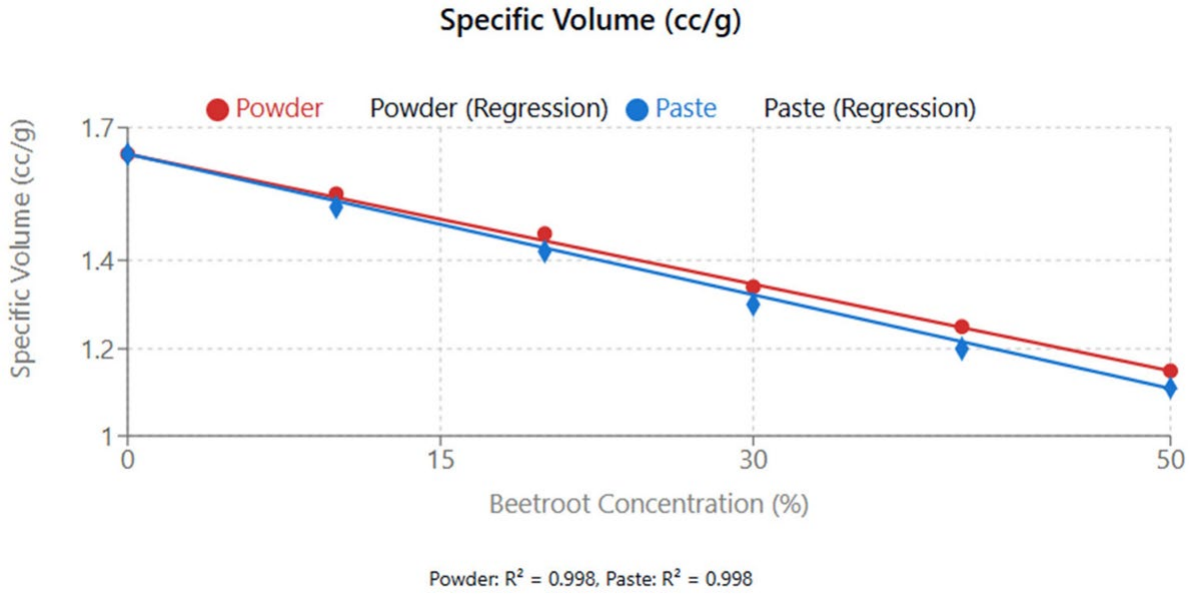
Effect of beetroot concentration (% w/w) on specific volume (mL/g) of cupcakes. Data points represent means ± standard deviations from three independent experiments (*n* = 3). Linear regression equations: powder formulation: *y* = −0.0098*x* + 1.64 (mL/g), *R* ^2^ = 0.998; paste formulation: *y* = −0.0106*x* + 1.64 (mL/g), *R* ^2^ = 0.998. Error bars indicate standard deviations.

Figure 7 shows a similar decreasing trend (*R* ^2^ > 0.99), reflecting the dilution of gluten proteins responsible for gas retention during baking. However, the weight of the cupcakes increased significantly (*p* < 0.05) with the increasing concentration of beetroot. This increase in weight can be attributed to the higher moisture retention capacity of beetroot fibers compared to wheat flour. The addition of fiber‐rich substitutes in wheat flour affects the gas retention capacity of baked goods, primarily due to gluten dilution. The type and concentration of dietary fiber can modify dough rheology and ultimately affect the quality of the final product. Substitution of gluten (1%–10%) has been reported to increase water absorption and alter dough stability and extensibility. These effects were evident when raw fiber from cocoa and apples was added to wheat‐based dough, and the bread properties were analyzed (Verbeke et al. 2024).

The observed relationship between beetroot concentration and specific volume is similar to findings by Struck et al. (2016), where the fiber‐rich ingredients compete with wheat flour for water, affecting gluten development and gas retention capacity. The slightly lower specific volume of paste formulations compared to powder at equivalent concentration levels can be attributed to differences in water distribution within the batter system. Paste forms of ingredients typically possess higher water‐binding capacity, which affects the availability of free water necessary for optimal gluten development (Foschia et al. 2013).

#### 3.1.3 Colourimetric Parameters and Visual Attributes

**FIGURE 8:**
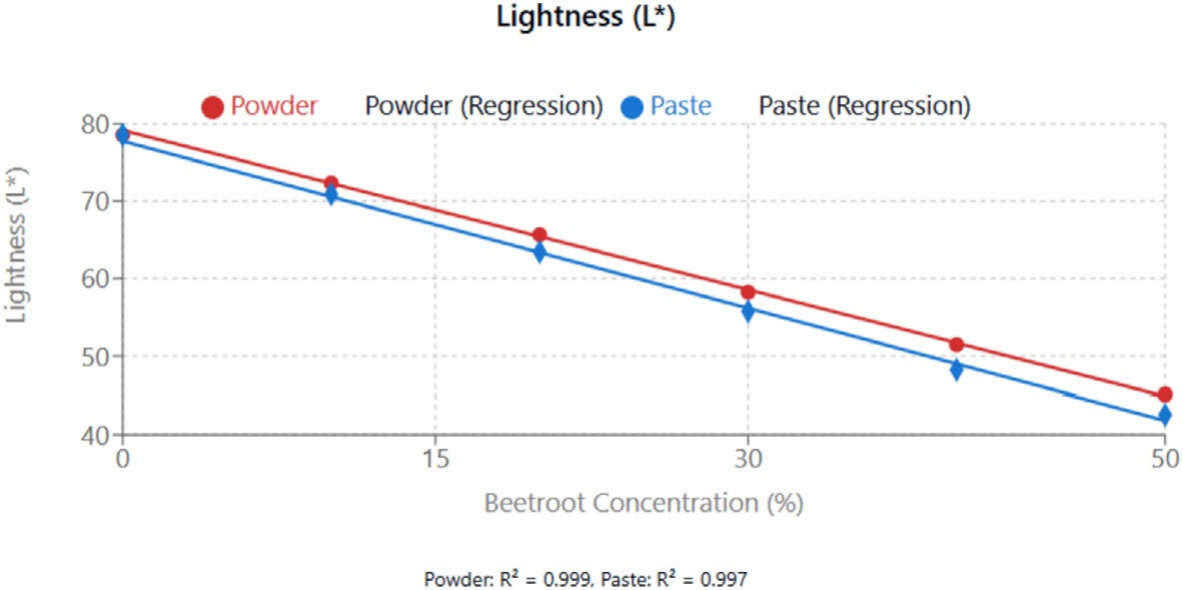
Effect of beetroot concentration (% w/w) on lightness (L*) of cupcakes. Data points represent means ± standard deviations from three independent experiments with three measurements per sample (*n* = 9). Linear regression equations: powder formulation: *y* = −0.687*x* + 79.160, *R* ^2^ = 0.999; paste formulation: *y* = −0.719*x* + 77.746, *R* ^2^ = 0.997. Error bars indicate standard deviations.

**FIGURE 9:**
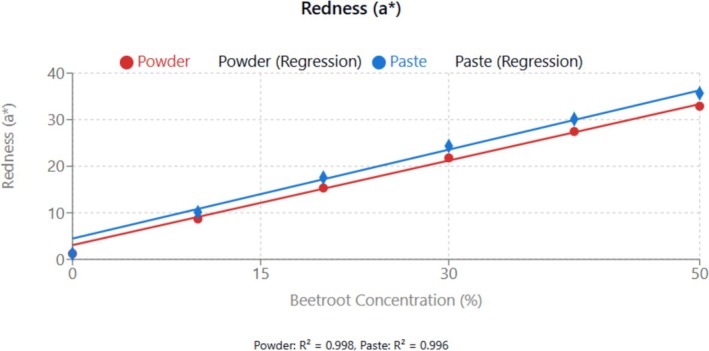
Effect of beetroot concentration (% w/w) on redness (a*) of cupcakes. Data points represent means ± standard deviations from three independent experiments with three measurements per sample (*n* = 9). Linear regression equations: powder formulation: *y* = 0.606*x* + 3.051, *R* ^2^ = 0.998; paste formulation: *y* = 0.637*x* + 4.464, *R* ^2^ = 0.996. Error bars indicate standard deviations.

**FIGURE 10:**
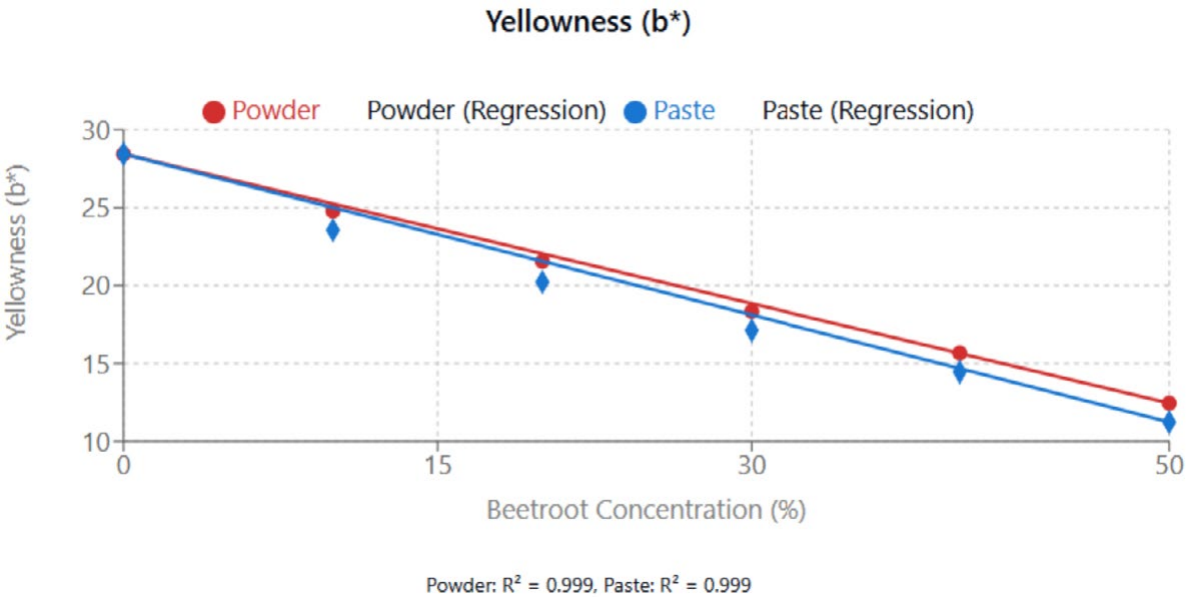
Effect of beetroot concentration (% w/w) on yellowness (b*) of cupcakes. Data points represent means ± standard deviations from three independent experiments with three measurements per sample (*n* = 9). Linear regression equations: Powder formulation: *y* = −0.320*x* + 28.450, *R* ^2^ = 0.999; Paste formulation: *y* = −0.344*x* + 28.450, *R* ^2^ = 0.999. Error bars indicate standard deviations.

The color parameters (L*, a*, b*) of the beetroot‐fortified cupcakes are displayed in Table 4 and the effect of beetroot incorporation on the various color parameters in Figures 8, 9, 10. The L* value, representing lightness, decreased significantly (*p* < 0.05) with increasing beetroot concentration, indicating that the cupcakes became darker. Conversely, the a* value, representing redness, increased significantly (*p* < 0.05) with increasing beetroot concentration, consistent with the characteristic red color of beetroot due to its betalain pigments. The b* value, representing yellowness, decreased significantly (*p* < 0.05) with increasing beetroot concentration. This decrease in yellowness could be attributed to the masking effect of the red beetroot pigments on the yellow pigments present in wheat flour and eggs (Janiszewska‐Turak et al. 2021).

**TABLE 4:** Color characteristics of beetroot‐fortified cupcakes.

Sample	L* (Lightness)	a* (Redness)	b* (Yellowness)
Control	78.56 ± 1.89ᵃ	1.23 ± 0.15ᵃ	28.45 ± 1.32ᵃ
BRP‐10%	72.34 ± 1.75ᵇ	8.67 ± 0.78ᵇ	24.78 ± 1.18ᵇ
BRP‐20%	65.67 ± 1.62ᶜ	15.32 ± 1.12ᶜ	21.56 ± 1.04ᶜ
BRP‐30%	58.23 ± 1.48ᵈ	21.78 ± 1.45ᵈ	18.34 ± 0.95ᵈ
BRP‐40%	51.45 ± 1.36ᵉ	27.45 ± 1.67ᵉ	15.67 ± 0.87ᵉ
BRP‐50%	45.12 ± 1.23ᶠ	32.89 ± 1.93ᶠ	12.45 ± 0.76ᶠ
BRT‐10%	70.89 ± 1.71ᵇ	10.12 ± 0.82ᵇ	23.56 ± 1.15ᵇᶜ
BRT‐20%	63.45 ± 1.57ᶜ	17.56 ± 1.25ᶜ	20.23 ± 1.01ᶜᵈ
BRT‐30%	55.78 ± 1.43ᵈᵉ	24.34 ± 1.53ᵈᵉ	17.12 ± 0.92ᵈᵉ
BRT‐40%	48.23 ± 1.32ᵉᶠ	30.12 ± 1.78ᵉᶠ	14.45 ± 0.84ᵉᶠ
BRT‐50%	42.56 ± 1.18ᶠᵍ	35.67 ± 2.05ᶠᵍ	11.23 ± 0.72ᶠᵍ

*Note:* The data shown represent means ± standard deviations from three replicated determinations. Values within the same columns marked with different superscript letters indicate statistically significant differences (*p* < 0.05).

The b* value, representing yellowness, decreased significantly (*p* < 0.05) with increasing beetroot concentration. This decrease in yellowness could be attributed to the masking effect of the red beetroot pigments on the yellow pigments present in wheat flour and eggs.

Lightness showed the decrease linearly as concentration increased. Paste formulations produced darker cupcakes than powder.

Redness (a*) increased dramatically with increasing beetroot concentration (*R* ^2^ > 0.99), reflecting the betalain pigment contribution. Paste formulations showed consistently higher redness values than powder.

Hue angle analysis revealed systematic color transition from yellow‐orange tones (control: 87.5°) to red‐orange coloration with increasing beetroot concentration. At maximum substitution levels, hue angles decreased to 20.7° (powder) and 17.4° (paste), indicating a significant shift toward pure red coloration. This transition reflects the progressive masking of wheat flour's natural yellow pigments by beetroot's characteristic betalain compounds.

Chroma analysis, representing color saturation intensity, showed interesting patterns. While initial substitution levels (10%–20% w/w) resulted in slight decreases in chroma due to color blending effects, higher concentrations (30%–50% w/w) led to progressive increases in color saturation. The maximum chroma values of 35.2 (powder) and 37.4 (paste) at 50% substitution exceeded the control (28.5), indicating that beetroot incorporation enhanced overall color vibrancy.

Form‐dependent color differences were evident throughout the concentration range. Paste formulations consistently exhibited higher a* values (redness) and chroma values compared to powder at equivalent concentrations, with differences becoming more pronounced at higher substitution levels. At 50% (w/w) substitution, paste showed 8.5% higher redness and 6.2% higher chroma than powder. This superiority reflects better preservation of heat‐sensitive betalain pigments in the paste form, where the natural matrix provides protective effects against thermal degradation during processing. The lower hue angles in paste formulations (averaging 2°–3° lower than powder) indicate purer red coloration, supporting the hypothesis that paste processing better maintains the original pigment profile of fresh beetroot compared to the thermal treatment required for powder production (Janiszewska‐Turak et al. 2021).

Interestingly, cupcakes fortified with beetroot paste exhibited slightly higher a* values and lower L* values compared to those with beetroot powder at equivalent substitution levels. This difference could be attributed to the different processing methods, with drying potentially causing some degradation of the betalain pigments in the beetroot powder. The superior color stability observed in paste formulations reflects principles demonstrated by Esmaeili et al. (2024) in their work on natural antioxidant preservation. Their findings on protective matrix effects against oxidative degradation support our hypothesis that paste preparation better preserves betalain pigments through maintained antioxidant synergism and water activity buffering.

The color effects of betalain‐rich ingredients in wheat‐based products depend on dosage and are affected by thermal processing, which may degrade these pigments (Azeredo 2009). The stability of betalains in less processed forms explains why paste formulations are valued differently compared to powders due to their high color retention property (Herbach et al. 2004). Various studies indicate that thermal treatment can modify the color profile of betalains, causing a transition from red to yellow‐orange shades due to betalain breakdown, with betacyanin degrading more slowly than betaxanthin (Sánchez‐Chávez et al. 2015). Prolonged heating can lead to significant color changes, as evidenced by red beet juice, where extended heating notably alters the hue and tone (Herbach et al. 2004).

Additionally, the presence of higher moisture content in the paste formulation may provide a protective effect against oxidation, as suggested by Herbach et al. (2004). Thus, the observed color difference between paste and powder formulations reflects both processing‐induced losses and matrix effects, with the paste form better preserving the natural pigments of beetroot.

### 3.2 Sensory Evaluation and Consumer Acceptance

The sensory attributes of the beetroot‐fortified cupcakes are presented in Table 5. All sensory parameters were significantly (*p* < 0.05) affected by the incorporation of beetroot, with the effect being more pronounced as the beetroot concentration increased.

**TABLE 5:** Sensory attributes of beetroot‐fortified cupcakes.

Sample	Color	Sponginess	Taste	Aroma	Mouthfeel	Appearance	Overall acceptability
Control	7.8 ± 0.7ᵃ	8.1 ± 0.6ᵃ	7.9 ± 0.8ᵃ	7.7 ± 0.7ᵃ	8.0 ± 0.5ᵃ	7.9 ± 0.6ᵃ	8.0 ± 0.7ᵃ
BRP‐10%	7.5 ± 0.8ᵃ	7.8 ± 0.7ᵃᵇ	7.7 ± 0.7ᵃᵇ	7.6 ± 0.6ᵃ	7.8 ± 0.6ᵃᵇ	7.7 ± 0.7ᵃᵇ	7.8 ± 0.6ᵃᵇ
BRP‐20%	8.2 ± 0.6ᵃ	7.6 ± 0.8ᵃᵇ	8.0 ± 0.6ᵃ	7.8 ± 0.7ᵃ	7.5 ± 0.7ᵃᵇ	8.1 ± 0.5ᵃ	8.2 ± 0.5ᵃ
BRP‐30%	7.3 ± 0.9ᵃᵇ	7.2 ± 0.9ᵇᶜ	7.4 ± 0.8ᵇᶜ	7.4 ± 0.8ᵃᵇ	7.1 ± 0.8ᵇᶜ	7.5 ± 0.8ᵃᵇ	7.4 ± 0.7ᵇᶜ
BRP‐40%	6.5 ± 1.0ᵇᶜ	6.7 ± 1.0ᶜ	6.8 ± 0.9ᶜ	6.9 ± 0.9ᵇᶜ	6.6 ± 0.9ᶜ	6.8 ± 0.9ᵇᶜ	6.7 ± 0.8ᶜ
BRP‐50%	5.7 ± 1.1ᶜᵈ	6.0 ± 1.1ᵈ	6.1 ± 1.0ᵈ	6.2 ± 1.0ᶜᵈ	5.9 ± 1.0ᵈ	6.0 ± 1.0ᶜᵈ	5.9 ± 0.9ᵈ
BRT‐10%	7.6 ± 0.7ᵃ	7.9 ± 0.6ᵃ	7.8 ± 0.7ᵃᵇ	7.7 ± 0.6ᵃ	7.9 ± 0.5ᵃ	7.8 ± 0.6ᵃᵇ	7.9 ± 0.6ᵃᵇ
BRT‐20%	8.0 ± 0.6ᵃ	7.7 ± 0.7ᵃᵇ	7.9 ± 0.6ᵃ	7.8 ± 0.7ᵃ	7.6 ± 0.7ᵃᵇ	7.9 ± 0.6ᵃ	8.0 ± 0.5ᵃ
BRT‐30%	8.3 ± 0.5ᵃ	7.5 ± 0.8ᵃᵇ	8.1 ± 0.5ᵃ	8.0 ± 0.6ᵃ	7.4 ± 0.7ᵃᵇ	8.2 ± 0.5ᵃ	8.3 ± 0.4ᵃ
BRT‐40%	7.1 ± 0.9ᵇ	7.0 ± 0.9ᵇᶜ	7.2 ± 0.8ᵇᶜ	7.3 ± 0.8ᵃᵇ	6.9 ± 0.8ᵇᶜ	7.2 ± 0.9ᵇ	7.2 ± 0.7ᵇᶜ
BRT‐50%	6.2 ± 1.0ᶜ	6.3 ± 1.0ᶜᵈ	6.5 ± 0.9ᶜᵈ	6.6 ± 0.9ᵇᶜ	6.2 ± 0.9ᶜᵈ	6.4 ± 1.0ᶜ	6.3 ± 0.8ᶜᵈ

*Note:* The data presented are means ± standard deviations from the scores of fifteen panelists. The values within the same column that have different superscripts are considered significantly different (*p* < 0.05). BRP stands for Beetroot Powder, while BRT refers to Beetroot Paste.

The successful development of consumer‐acceptable functional products requires careful optimization of multiple parameters simultaneously. Amiri et al. (2025) emphasized the importance of systematic optimization approaches in functional food development, supporting our methodology of using multiple concentration levels and forms to identify successful incorporation strategies.

### 3.3 Multivariate Analysis of Physical Properties

The cupcakes of 20% (w/w) beetroot powder and 30% beetroot paste received the highest acceptability scores (8.2 and 8.3 out of 9, respectively), slightly surpassing the control. Beetroot paste formulations were consistently most preferred over powder formulations at equivalent substitution levels.

**FIGURE 11:**
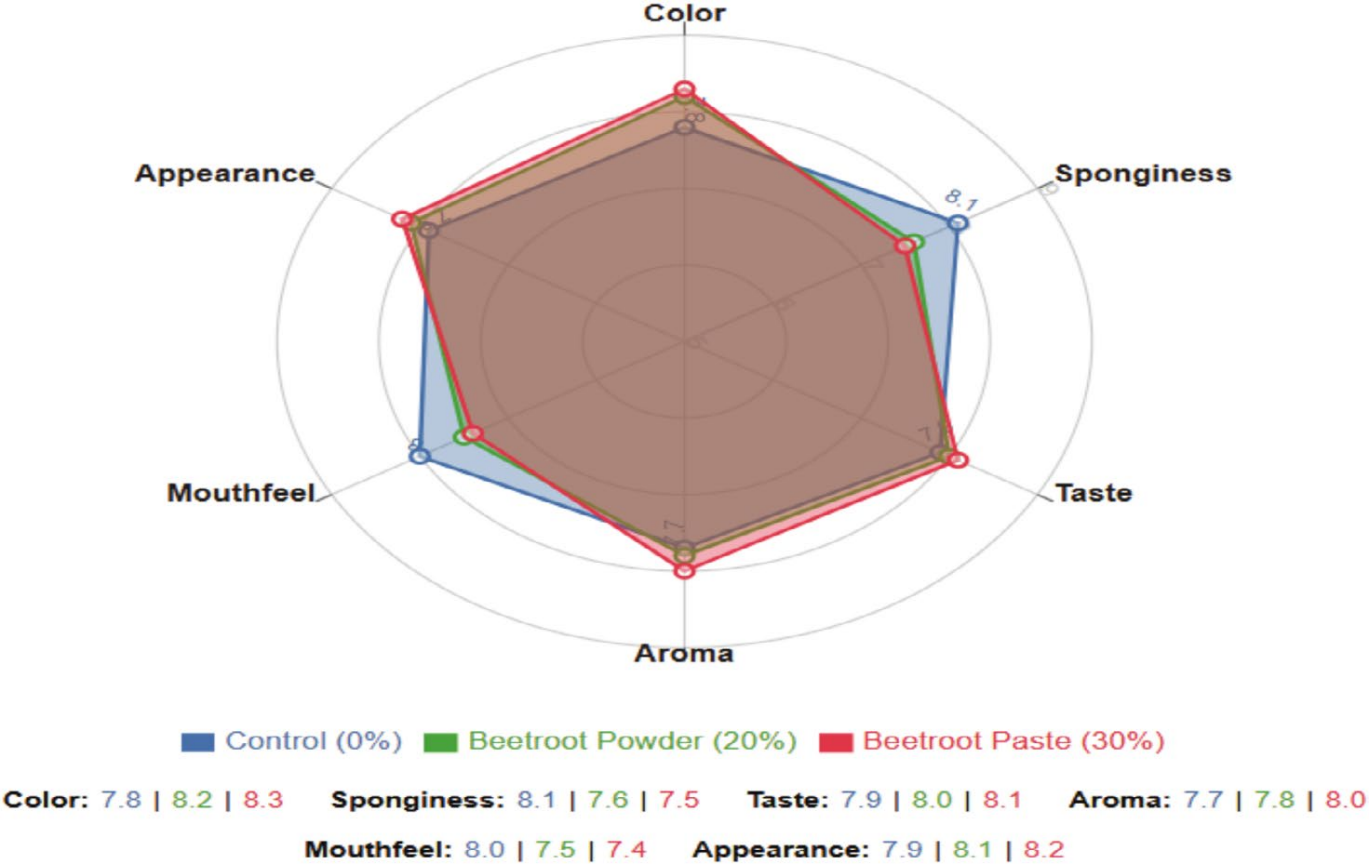
Sensory profile comparison of control cupcakes (0% beetroot), preferred beetroot powder‐fortified cupcakes (20% w/w), and optimal beetroot paste‐fortified cupcakes (30% w/w). Values represent mean hedonic ratings on a 9‐point scale (*n* = 15).

Figure 11 illustrates that the formulations (20% powder and 30% paste) maintained or enhanced key sensory attributes compared to the control. The 30% paste formulation showed improvements in color, taste, and appearance compared to the powder, while underperforming in terms of sponginess and mouthfeel. The scores of these formulations were not significantly different from the control (*p* > 0.05) for most of the attributes, as indicated in Table 5. This affirms that beetroot can be incorporated at these levels without negatively impacting sensory acceptance.

The higher acceptance of paste over powder at concentrations above 30% was statistically significant (*p* < 0.05) for overall acceptability and most attributes. This may be attributed to the better integration of beetroot paste into the cupcake matrix, resulting in a more uniform distribution and a less pronounced beetroot flavor.

The identification of 20% and 30% as successful incorporation levels parallels findings by Hussain et al. (2006), who reported acceptance thresholds between 15% and 30% for natural ingredients in pastry products. The higher acceptance of paste compared to powder supports the observation by Lu et al. (2010), where wet ingredients showed better sensory integration compared to dry ones in similar applications. While beetroot paste formulations are preferred, it is essential to acknowledge that some consumers may favor traditional flavor and texture, which could influence overall acceptability.

**FIGURE 12:**
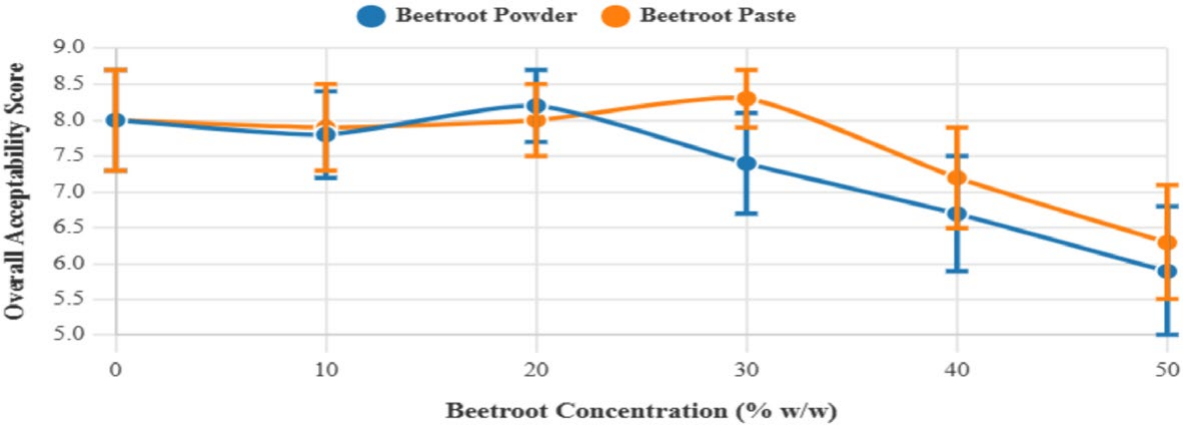
Changes in overall acceptability scores with increasing beetroot concentration (%) for both powder and paste formulations. Values represent mean hedonic ratings on a 9‐point scale. Error bars indicate standard deviations (*n* = 15).

The overall acceptability trend (Figure 12) demonstrates that beetroot can be successfully incorporated up to certain thresholds before acceptability declines. The inflection points occur at 20% for powder and 30% for paste, suggesting that paste allows higher beetroot incorporation while maintaining sensory quality.

**FIGURE 13:**
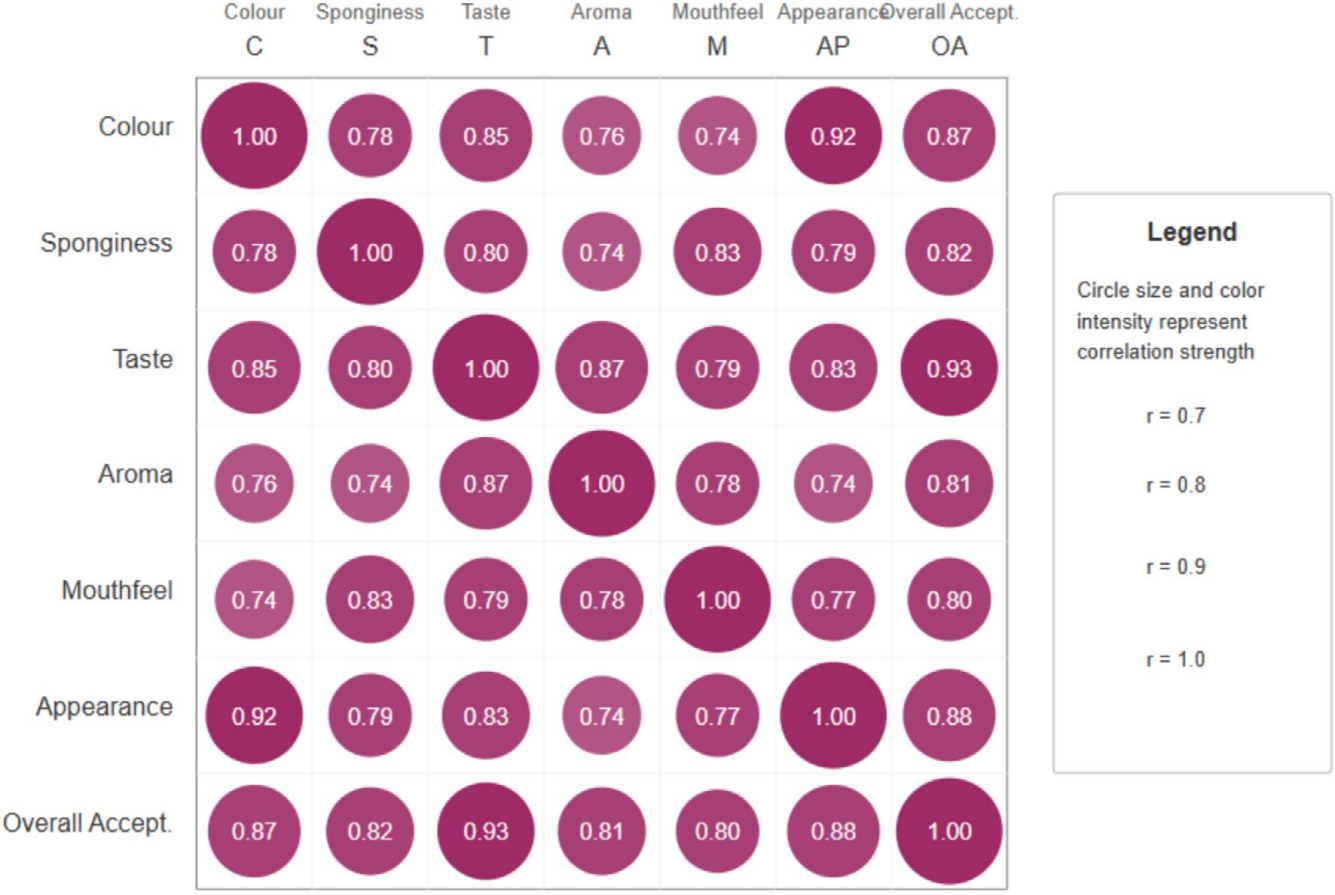
Correlation matrix of the sensory attributes of beetroot‐fortified cupcakes.

It was noted in Figure 13 that all correlations in this study were positive, reflecting the cohesive nature of the sensory profile. Such uniformly high correlations are typically unusual in sensory studies. However, these findings suggest that panelists may have placed more emphasis on taste and appearance when assessing overall acceptability, indicating that the appealing visual characteristics of beetroot cupcakes are crucial and likely to enhance taste perception.

Beetroot affects all sensory attributes in a coordinated manner, rather than affecting specific aspects independently. This means product developers cannot easily modify one sensory aspect without impacting the others. This result also shows that beetroot concentration affects all attributes simultaneously. This explains why there are clear optimal concentration points (20% for powder and 30% for paste) beyond which all attributes decline together.

#### 3.3.1 Principal Component Analysis and Variable Relationships

**FIGURE 14:**
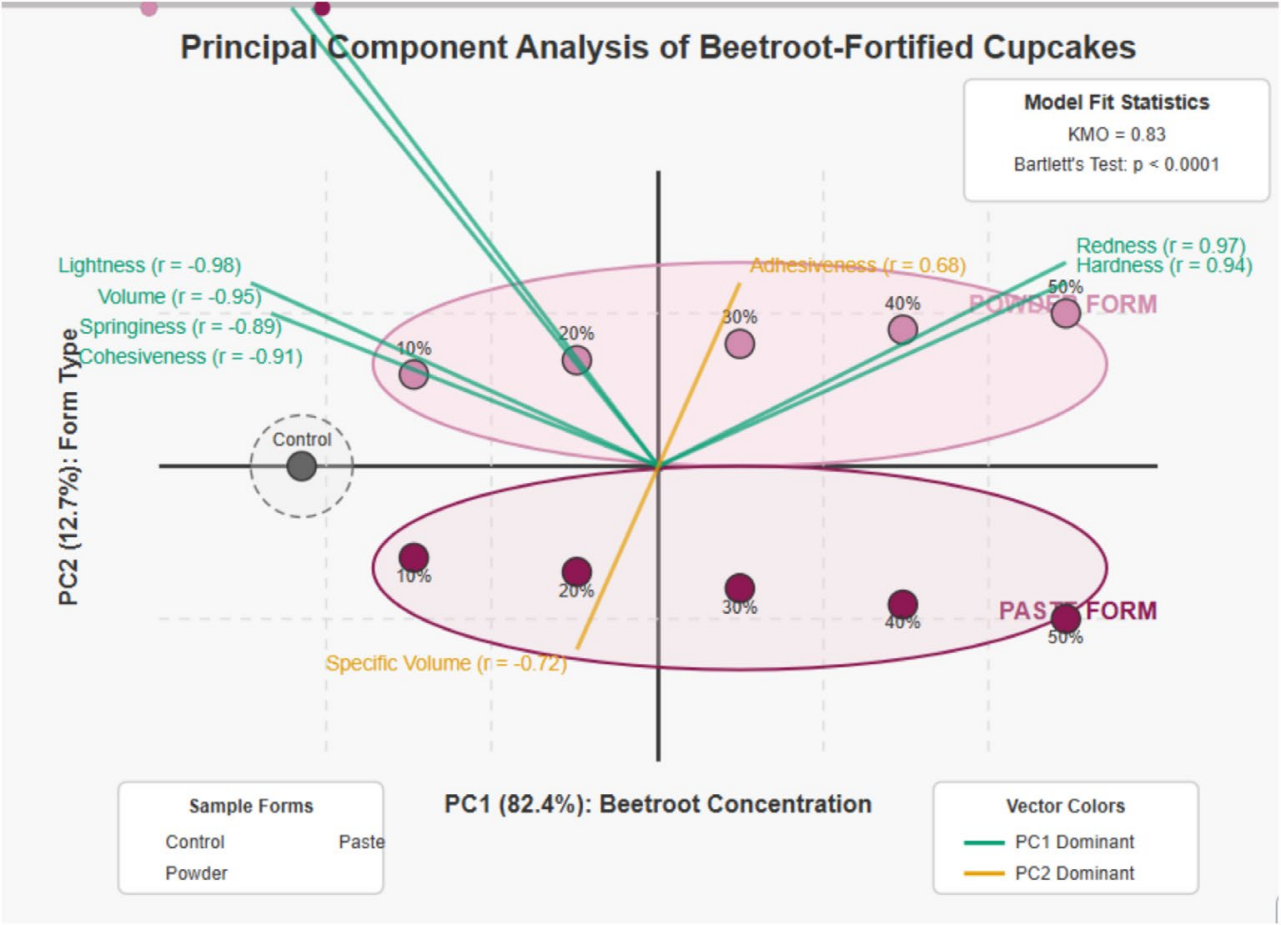
Principal component analysis (PCA) biplot of physical properties in beetroot‐fortified cupcakes. PC1 (82.4% variance) corresponds to beetroot concentration, while PC2 (12.7% variance) differentiates between powder and paste formulations. Eigenvalues for the first three principal components were 6.6, 1.0, and 0.4, respectively, with PC1 and PC2 explaining 95.1% of total variance.

Principal Component 1 (horizontal axis) strongly correlates with beetroot concentration, accounting for 82.4% of the data variance. Points progress from left (lower concentration) to right (higher concentration). Principal Component 2 (vertical axis) separates powder formulations (positive values) from paste formulations (negative values), accounting for 12.7% of variance. Figure 14 demonstrates that beetroot concentration is the primary factor influencing physical properties, with formulation type (powder vs. paste) being a significant secondary factor. This aligns with work done by Nollet et al. (2012), where it was concluded that the ingredient concentration of a food system accounts for the majority of variance in physical properties, with processing variables accounting for the secondary influence.

Further analysis of the PCA loadings reveals important correlations between specific properties and principal components. PC1 shows strong positive correlations with hardness (*r* = 0.94) and redness (a*) (*r* = 0.97), and strong negative correlations with lightness (L*) (*r* = −0.98), volume (*r* = −0.95), springiness (*r* = −0.89), and cohesiveness (*r* = −0.91). This confirms that the concentration of beetroot simultaneously affects multiple physical properties predictably. PC2 shows moderate correlations with adhesiveness (*r* = 0.68) and specific volume (*r* = −0.72), differentiating between powder and paste formulations. The orthogonal relationship between these components indicates that concentration and form operate as independent factors, suggesting potential for separate optimization in product development.

## 4 Conclusion

The present study shows the feasibility of incorporating beetroot as a functional ingredient in cupcakes. The physical properties of beetroot‐fortified cupcakes exhibited strong dose‐dependent relationships with concentration levels, enabling the development of predictive mathematical models that can serve as valuable tools for food product developers. The form of beetroot significantly influenced the properties of the final product, with paste consistently outperforming powder in maintaining desirable textural attributes and achieving superior color development.

The acceptable concentrations identified through sensory evaluation (20% for powder and 30% for paste) represent practical levels at which beetroot can be incorporated without compromising consumer acceptability. These levels allow for significant nutritional enhancement while maintaining or even improving certain sensory attributes compared to the control. The higher acceptable concentration of paste (30%) compared to powder (20%) suggests that paste is a more effective form for maximizing functional benefits in cupcake formulations.

Principal Component Analysis provided valuable insights into the interrelationships between various physical properties, confirming that beetroot concentration was the primary factor influencing all measured parameters, with the form (powder vs. paste) being a significant secondary factor. The identified regression models offer practical tools for predicting specific properties at different concentration levels, enabling precise formulation adjustments.

While this study establishes the sensory and physicochemical feasibility of beetroot incorporation at practical levels that maintain consumer acceptability, comprehensive nutritional analysis is warranted to fully validate the functional benefits and establish evidence‐based optimal incorporation levels for health claims. The determination of acceptable beetroot concentrations in this study was based primarily on sensory acceptability and physicochemical properties. Complete nutritional characterization of these formulations, including proximate composition, mineral content, vitamin levels, betalain quantification, and antioxidant activity assessment, represents essential complementary research to support health‐related claims and establish true nutritional optimization parameters.

Future research should investigate the specific nutritional enhancements achieved at these acceptable concentrations, the impact of storage on color stability and texture, and the potential health benefits of regular consumption of these functional cupcakes. Additionally, the application of beetroot in other bakery products and the development of strategies to further increase acceptable incorporation levels warrant exploration.

This study contributes to addressing healthier bakery options and also offers a potential solution to post‐harvest losses of beetroot in Ghana. The successful development of beetroot‐fortified cupcakes with acceptable quality attributes demonstrates the potential for transforming traditional indulgence foods into vehicles for delivering beneficial bioactive compounds.

## Author Contributions

**Berlinda Agyei‐Poku:** conceptualization (equal), data curation (equal), funding acquisition (equal), investigation (equal). **Sakyiwaa Afia Amponsah:** data curation (equal), formal analysis (equal), investigation (equal), methodology (equal), software (equal), visualization (equal), writing – original draft (equal), writing – review and editing (equal). **Dedo Doreen Adi:** conceptualization (equal), data curation (equal), formal analysis (equal), project administration (equal), supervision (equal), writing – review and editing (equal). **Barikisu Mohammed:** investigation (equal), methodology (equal), resources (equal), visualization (equal), writing – review and editing (equal).

## Conflicts of Interest

The authors declare no conflicts of interest.

## Data Availability

Data is available upon request from the corresponding author.

## Appendix A Comprehensive Mathematical Models

### Physical Property Prediction Models

[Table fsn370863-tbl-0006]

**TABLE A1 fsn370863-tbl-0006:** Complete regression equations for physical properties.

Property	Form	Regression equation	*R* ^2^	RMSE	*p*	95% CI slope
Hardness (N)	Powder	*H* = 0.304C + 15.795	0.990	0.52	< 0.001	[0.286, 0.322]
Hardness (N)	Paste	*H* = 0.256C + 15.508	0.976	0.48	< 0.001	[0.235, 0.277]
Springiness	Powder	*S* = −0.0036C + 0.922	0.968	0.015	< 0.001	[−0.0041, −0.0031]
Springiness	Paste	*S* = −0.0029C + 0.917	0.955	0.013	< 0.001	[−0.0034, −0.0024]
Cohesiveness	Powder	Coh = −0.0048C + 0.790	0.979	0.019	< 0.001	[−0.0054, −0.0042]
Cohesiveness	Paste	Coh = −0.0039C + 0.784	0.970	0.017	< 0.001	[−0.0045, −0.0033]
Volume (mL)	Powder	V = −0.362C + 86.208	0.998	0.89	< 0.001	[−0.373, −0.351]
Volume (mL)	Paste	V = −0.375C + 85.033	0.998	0.92	< 0.001	[−0.387, −0.363]
Specific Volume (mL/g)	Powder	SV = −0.0098C + 1.640	0.998	0.021	< 0.001	[−0.0101, −0.0095]
Specific Volume (mL/g)	Paste	SV = −0.0106C + 1.640	0.998	0.023	< 0.001	[−0.0110, −0.0102]
Lightness (*L**)	Powder	*L** = −0.687C + 79.160	0.999	0.42	< 0.001	[−0.695, −0.679]
Lightness (*L**)	Paste	*L** = −0.719C + 77.746	0.997	0.48	< 0.001	[−0.730, −0.708]
Redness (*a**)	Powder	*a** = 0.606C + 3.051	0.998	0.35	< 0.001	[0.598, 0.614]
Redness (*a**)	Paste	*a** = 0.637C + 4.464	0.996	0.41	< 0.001	[0.627, 0.647]
Yellowness (*b**)	Powder	*b** = −0.320C + 28.450	0.999	0.18	< 0.001	[−0.325, −0.315]
Yellowness (*b**)	Paste	*b** = −0.344C + 28.450	0.999	0.19	< 0.001	[−0.350, −0.338]

*Note:* Where C = beetroot concentration (% w/w).

Abbreviations: CI = confidence interval; RMSE = root mean square error.

### Model Validation and Assumptions

Regression assumptions verification:

- Linearity: Confirmed through residual vs. fitted plots showing random scatter
- Independence: Verified through Durbin–Watson test (*d* = 1.85–2.15 for all models)
- Homoscedasticity: Confirmed via Breusch–Pagan test (*p* > 0.05 for all models)
- Normality: Verified through Shapiro–Wilk test on residuals (*p* > 0.05 for all models)

Cross‐validation results:

**Table:**

Property	Form	Leave‐one‐out CV *R* ^2^	10‐Fold CV *R* ^2^	Prediction Error (MAPE)
Hardness	Powder	0.985	0.987	2.8%
Hardness	Paste	0.970	0.973	3.1%
Volume	Powder	0.996	0.997	1.2%
Volume	Paste	0.996	0.997	1.3%
Lightness	Powder	0.998	0.998	0.8%
Lightness	Paste	0.995	0.996	1.1%

Abbreviation: MAPE = Mean Absolute Percentage Error.

### Predictive Applications

Formulation optimization calculator:

For any desired target property value (*Y*), the required beetroot concentration can be calculated using:

$$C=\left(Y–b_{0}\right)/b_{1}$$

where *C* = required concentration (% w/w); *Y* = target property value; *b*_0_ = y‐intercept; *b*_1_ = slope coefficient.

Example calculations:

To achieve hardness of 25 N using powder:

$$C=\left(25–15.795\right)/0.304=30.3\%\left(w/w\right)$$

To achieve *L* value of 55 using paste:*

$$C=\left(55–77.746\right)/\left(-0.719\right)=31.6\%\left(w/w\right)$$

#### Quality Control Limits

[Table fsn370863-tbl-0007]

**TABLE A2 fsn370863-tbl-0007:** Acceptable ranges for physical properties.

Property	Acceptable range	Powder conc. range	Paste conc. range
Hardness (N)	18–28	7.3%–40.1%	9.8%–48.8%
Springiness	0.75–0.92	0.6%–47.2%	0%–57.6%
Volume (mL)	68–85	3.3%–50.8%	0%–45.4%
*L** (Lightness)	45–78	1.7%–48.4%	0%–45.5%
Overall Accept.	≥ 7.0	0%–35%	0%–45%

#### Statistical Power Analysis

Sample size adequacy:

Post hoc power analysis confirmed adequate sample sizes for detecting meaningful differences:

- Effect size (Cohen's *d*): 0.8–1.2 for physical properties
- Statistical power (1 − *β*): 0.85–0.95
- Alpha level (*α*): 0.05
- Minimum detectable difference: 5%–10% relative change

Sensitivity analysis:

The models demonstrate robust predictive capability within the tested concentration range (0%–50% w/w). Extrapolation beyond this range is not recommended without additional experimental validation.

#### Comparative Analysis: Powder vs. Paste

Rate of change comparison (% change per 1% beetroot addition):

**Table:**

Property	Powder rate	Paste rate	Difference	Statistical significance
Hardness	+1.67%/%	+1.41%/%	0.26%/%	*p* < 0.001
Springiness	−0.39%/%	−0.32%/%	0.07%/%	*p* < 0.05
Volume	−0.42%/%	−0.44%/%	0.02%/%	*p* > 0.05
Lightness	−0.87%/%	−0.92%/%	0.05%/%	*p* < 0.05
Redness	+49.3%/%	+14.3%/%	35.0%/%	*p* < 0.001

## References

[fsn370863-bib-0001] Agrahar‐Murugkar, D. , A. Zaidi , N. Kotwaliwale , and C. Gupta . 2016. “Effect of Egg‐Replacer and Composite Flour on Physical Properties, Color, Texture and Rheology, Nutritional and Sensory Profile of Cakes.” Journal of Food Quality 39, no. 5: 425–435. 10.1111/jfq.12224.

[fsn370863-bib-0002] Aishah, B. , J. Fadhilah , A. Noriham , A. Noorlaila , L. Aisyah Jacklin , and F. E. Yun Irma . 2020. “Reformulation of Le'Natura® Biscuit: Effects on Textural, Sensorial, Nutritional and Glycemic Index Values.” International Pharmacy Acta 3, no. 1: e4. 10.22037/ipa.v3i1.31577.

[fsn370863-bib-0003] Amiri, M. , B. Hassani , H. Babapour , et al. 2025. “Optimization of Enzyme Hydrolysis to Improve Functional and Structural Properties of Microalgae Protein Extract.” Journal of Food Science 90, no. 4: e70129. 10.1111/1750-3841.70129. 40184030

[fsn370863-bib-0004] Asadi, S. Z. , and M. A. Khan . 2021. “The Effect of Beetroot (*Beta vulgaris* L.) Leaves Powder on Nutritional, Textural, Sensorial and Antioxidant Properties of Cookies.” Journal of Culinary Science & Technology 19, no. 5: 424–438. 10.1080/15428052.2020.1787285.

[fsn370863-bib-0005] Azeredo, H. M. 2009. “Betalains: Properties, Sources, Applications, and Stability–A Review.” International Journal of Food Science and Technology 44, no. 12: 2365–2376.

[fsn370863-bib-0006] Beikzadeh, S. , A. Khezerlou , S. M. Jafari , Z. Pilevar , and A. M. Mortazavian . 2020. “Seed Mucilages as the Functional Ingredients for Biodegradable Films and Edible Coatings in the Food Industry.” Advances in Colloid and Interface Science 280: 102164. 10.1016/j.cis.2020.102164. 32335381

[fsn370863-bib-0007] Chhikara, N. , K. Kushwaha , P. Sharma , Y. Gat , and A. Panghal . 2019. “Bioactive Compounds of Beetroot and Utilization in Food Processing Industry: A Critical Review.” Food Chemistry 272: 192–200. 30309532 10.1016/j.foodchem.2018.08.022

[fsn370863-bib-0008] Clifford, T. , G. Howatson , D. J. West , and E. J. Stevenson . 2015. “The Potential Benefits of Red Beetroot Supplementation in Health and Disease.” Nutrients 7, no. 4: 2801–2822. 25875121 10.3390/nu7042801 PMC4425174

[fsn370863-bib-0009] Djordjević, M. , M. Djordjević , D. Šoronja‐Simović , I. Nikolić , and Z. Šereš . 2021. “Delving Into the Role of Dietary Fiber in Gluten‐Free Bread Formulations: Integrating Fundamental Rheological, Technological, Sensory, and Nutritional Aspects.” Polysaccharides 3, no. 1: 59–82.

[fsn370863-bib-0010] Ergönül, B. 2013. “Consumer Awareness and Perception to Food Safety: A Consumer Analysis.” Food Control 32, no. 2: 461–471. 10.1016/j.foodcont.2013.01.018.

[fsn370863-bib-0011] Esmaeili, F. , M. Mehrabi , H. Babapour , B. Hassani , and A. Abedinia . 2024. “Active Coating Based on Carboxymethyl Cellulose and Flaxseed Mucilage, Containing Burdock Extract, for Fresh‐Cut and Fried Potatoes.” LWT 192: 115726. 10.1016/j.lwt.2024.115726.

[fsn370863-bib-0012] Flores‐Mancha, M. A. , M. G. Ruíz‐Gutiérrez , A. L. Rentería‐Monterrubio , et al. 2021. “Stirred Yogurt Added With Beetroot Extracts as an Antioxidant Source: Rheological, Sensory, and Physicochemical Characteristics.” Journal of Food Processing and Preservation 45, no. 7: 15628. 10.1111/jfpp.15628.

[fsn370863-bib-0013] Foschia, M. , D. Peressini , A. Sensidoni , and C. S. Brennan . 2013. “The Effects of Dietary Fibre Addition on the Quality of Common Cereal Products.” Journal of Cereal Science 58, no. 2: 216–227.

[fsn370863-bib-0014] Fraqueza, M. J. , and A. S. Barreto . 2014. “HACCP: Hazard Analysis and Critical Control Points.” In Handbook of Fermented Meat and Poultry, 469–485. Wiley.

[fsn370863-bib-0015] Gasparre, N. , A. Pasqualone , M. Mefleh , and F. Boukid . 2022. “Nutritional Quality of Gluten‐Free Bakery Products Labeled Ketogenic and/or Low‐Carb Sold in the Global Market.” Food 11, no. 24: 4095. 10.3390/foods11244095. PMC9778343 36553837

[fsn370863-bib-0016] Guerrero, M. , A. K. Stone , R. Singh , Y. C. Lui , F. Koksel , and M. T. Nickerson . 2025. “Effect of Extrusion Conditions on the Characteristics of Texturized Vegetable Protein From a Faba Bean Protein Mix and Its Application in Vegan and Hybrid Burgers.” Food 14, no. 4: 547. 10.3390/foods14040547 PMC11854062 40001991

[fsn370863-bib-0017] Herbach, K. M. , F. C. Stintzing , and R. Carle . 2004. “Impact of Thermal Treatment on Color and Pigment Pattern of Red Beet (*Beta vulgaris* L.) Preparations.” Journal of Food Science 69, no. 6: C491–C498. 10.1111/j.1365-2621.2004.tb10994.x.

[fsn370863-bib-0018] Herranz, B. , W. Canet , M. J. Jiménez , R. Fuentes , and M. D. Alvarez . 2016. “Characterisation of Chickpea Flour‐Based Gluten‐Free Batters and Muffins With Added Biopolymers: Rheological, Physical and Sensory Properties.” International Journal of Food Science and Technology 51, no. 5: 1087–1098.

[fsn370863-bib-0019] Hussain, S. , F. M. Anjum , M. S. Butt , M. I. Khan , and A. Asghar . 2006. “Physical and Sensoric Attributes of Flaxseed Flour Supplemented Cookies.” Turkish Journal of Biology 30, no. 2: 87–92.

[fsn370863-bib-0020] Janiszewska‐Turak, E. , K. Rybak , E. Grzybowska , E. Konopka , and D. Witrowa‐Rajchert . 2021. “The Influence of Different Pretreatment Methods on Color and Pigment Change in Beetroot Products.” Molecules 26, no. 12: 3683. 34208715 10.3390/molecules26123683 PMC8235720

[fsn370863-bib-0021] Khajeh, N. , H. Babapour , B. Hassani , A. Mohammadi Nafchi , L. Nouri , and A. Abedinia . 2025. “Effect of Zedo Gum‐Based Coatings Containing Tarragon and Zataria Multiflora Boiss Essential Oils on Oil Uptake, Acrylamide Formation and Physicochemical Properties of Fried Potato Strips.” Food Science & Nutrition 13, no. 6: e70347. 40444120 10.1002/fsn3.70347 PMC12121445

[fsn370863-bib-0022] Krapivnytska, I. , V. Ladyka , M. Ianchyk , S. Omelchenko , O. Melnyk , and F. Pertsevyi . 2022. “Scientific and Practical Aspects of Pectin and Pectin Products.”

[fsn370863-bib-0023] Lawless, H. T. , and H. Heymann . 2010. Sensory Evaluation of Food: Principles and Practices. Springer Science & Business Media.

[fsn370863-bib-0024] Liu, J. , J. Bi , D. J. McClements , et al. 2020. “Impacts of Thermal and Non‐Thermal Processing on Structure and Functionality of Pectin in Fruit‐and Vegetable‐Based Products: A Review.” Carbohydrate Polymers 250: 116890. 33049879 10.1016/j.carbpol.2020.116890

[fsn370863-bib-0025] Lu, T.‐M. , C.‐C. Lee , J.‐L. Mau , and S.‐D. Lin . 2010. “Quality and Antioxidant Property of Green Tea Sponge Cake.” Food Chemistry 119, no. 3: 1090–1095.

[fsn370863-bib-0026] Macdougall, D. B. 2010. “Colour Measurement of Food: Principles and Practice.” In Colour Measurement, 312–342. Elsevier. 10.1533/9780857090195.2.312.

[fsn370863-bib-0027] Masoodi, F. , B. Sharma , and G. Chauhan . 2002. “Use of Apple Pomace as a Source of Dietary Fiber in Cakes.” Plant Foods for Human Nutrition 57: 121–128. 12049144 10.1023/a:1015264032164

[fsn370863-bib-0028] Mattar, H. H. 2019. The Impact of Baked Food Matrices on Structural and Allergenic Properties of Food Allergens. University of Manchester (United Kingdom).

[fsn370863-bib-0029] Mensah, D. O. , A. R. Nunes , T. Bockarie , R. Lillywhite , and O. Oyebode . 2021. “Meat, Fruit, and Vegetable Consumption in Sub‐Saharan Africa: A Systematic Review and Meta‐Regression Analysis.” Nutrition Reviews 79, no. 6: 651–692. 10.1093/nutrit/nuaa032. 32556305

[fsn370863-bib-0030] Nollet, L. M. L. , F. ToldrÃ¡ , G. Paliyath , S. Benjakul , and Y. H. Hui . 2012. Food Biochemistry and Food Processing. John Wiley & Sons.

[fsn370863-bib-0031] Prokopov, T. , Z. Goranova , M. Baeva , A. Slavov , and C. M. Galanakis . 2015. “Effects of Powder From White Cabbage Outer Leaves on Sponge Cake Quality.” International Agrophysics 29, no. 4: 493–500. 10.1515/intag-2015-0055.

[fsn370863-bib-0032] Rosell, C. M. , J. A. Rojas , and C. Benedito De Barber . 2001. “Influence of Hydrocolloids on Dough Rheology and Bread Quality.” Food Hydrocolloids 15, no. 1: 75–81. 10.1016/S0268-005X(00)00054-0.

[fsn370863-bib-0033] Saeki, H. , N. Hettiarachchy , K. Sato , and M. Marshall . 2012. “Protein–saccharide interaction.” In Food Proteins and Peptides: Chemistry, Functionality, Interactions, and Commercialization, 230–253. CPS Press.

[fsn370863-bib-0034] Sánchez‐Chávez, W. , J. Cortez‐Arredondo , M. Solano‐Cornejo , and J. Vidaurre‐Ruiz . 2015. “Kinetics of Thermal Degradation of Betacyanins, Betaxantins and Vitamin C in a Juice‐Based Drink Beet (*Beta vulgaris* L.) and Honey.” Scientia Agropecuaria 6: 111–118. 10.17268/sci.agropecu.2015.02.03.

[fsn370863-bib-0035] Selahvarzi, A. , Y. Ramezan , M. R. Sanjabi , H. Mirsaeedghazi , F. Azarikia , and A. Abedinia . 2021. “Investigation of Antimicrobial Activity of Orange and Pomegranate Peels Extracts and Their Use as a Natural Preservative in a Functional Beverage.” Journal of Food Measurement and Characterization 15, no. 6: 5683–5694. 10.1007/s11694-021-01141-z.

[fsn370863-bib-0037] Soleimani, M. , N. Amini , B. Sadeghian , D. Wang , and L. Fang . 2018. “Heavy Metals and Their Source Identification in Particulate Matter (PM2.5) in Isfahan City, Iran.” Journal of Environmental Sciences 72: 166–175. 10.1016/j.jes.2018.01.002. 30244743

[fsn370863-bib-0038] Soutelino, M. E. M. , D. B. Da Silva , R. Da Silva Rocha , et al. 2023. “Yogurt Added With Beetroot Extract: Physicochemical Parameters, Biological Activities and Sensory Evaluation by Check‐All‐That‐Apply Method.” International Journal of Food Science & Technology 58, no. 6: 3303–3309. 10.1111/ijfs.16214.

[fsn370863-bib-0039] Struck, S. , L. Gundel , S. Zahn , and H. Rohm . 2016. “Fiber Enriched Reduced Sugar Muffins Made From Iso‐Viscous Batters.” LWT 65: 32–38. 10.1016/j.lwt.2015.07.053.

[fsn370863-bib-0040] Verbeke, C. , E. Debonne , S. Versele , F. Van Bockstaele , and M. Eeckhout . 2024. “Technological Evaluation of Fiber Effects in Wheat‐Based Dough and Bread.” Food 13, no. 16: 16. 10.3390/foods13162582. PMC11353414 39200509

[fsn370863-bib-0041] Wang, S. , Y. Fang , Y. Xu , et al. 2022. “The Effects of Different Extraction Methods on Physicochemical, Functional and Physiological Properties of Soluble and Insoluble Dietary Fiber From *Rubus chingiiHu*. Fruits.” Journal of Functional Foods 93: 105081. 10.1016/j.jff.2022.105081.

